# Deep eutectic solvent-driven surface activation of montmorillonite enhances heavy metal uptake and electrochemical charge-transfer properties

**DOI:** 10.1039/d6ra03601e

**Published:** 2026-09-08

**Authors:** Bukar Lawan, Abubakar Shettima Kubri

**Affiliations:** a Department of Chemical Engineering, University of Maiduguri P.M.B 1069 Maiduguri Borno State Nigeria baborlawan@gmail.com; b School of Chemical Engineering, Newcastle University NE1 7RU UK

## Abstract

Montmorillonite clay was modified using a zinc chloride–urea metal salt-based deep eutectic solvent (MSDES) to probe how DES-induced surface reconfiguration governs metal-ion coordination and interfacial behaviour. XRD and FTIR confirmed retention of the 2 : 1 aluminosilicate framework and Si–O–Si bands, with redistribution of surface hydroxyl groups indicating surface-level rather than structural modification. XPS quantified Zn 2p at 0.74 atomic% on the DES-modified surface against no detectable signal on the unmodified clay, confirming zinc incorporation. N_2_ physisorption showed a 61% increase in BET surface area (42.3 to 68.1 m^2^ g^−1^) and a 71% gain in total pore volume. Zeta potential remained negative across the working pH range, with the point of zero charge shifting from pH 2.5 to 3.2. Batch adsorption in real contaminated water under competitive multi-ion conditions gave a selectivity hierarchy of Pb^2+^ > Cr^3+^ > Cd^2+^. Single-component Langmuir capacities were 0.696, 0.154, and 0.105 mg g^−1^ for Pb^2+^, Cr^3+^, and Cd^2+^, 158%, 927%, and 163% higher than unmodified montmorillonite. Non-linear kinetics revealed distinct rate-determining behaviour. Pb^2+^ followed pseudo-second-order kinetics indicative of chemisorption, Cr^3+^ followed pseudo-first-order kinetics, and Cd^2+^ showed rapid uptake followed by progressive displacement. Capacity retention exceeded 87% for Pb^2+^ and 68% for Cd^2+^ across five regeneration cycles. DES treatment reduced charge-transfer resistance from 2850 to 620 Ω and raised anodic peak current by 235% relative to unmodified montmorillonite. These results link DES-induced surface reconfiguration to the metal-stabilising and electrochemical properties of the material, supporting its use as a clay-based scaffold for catalytic and remediation applications.

## Abstract 2

ZnCl_2_–urea DES treatment raises montmorillonite BET surface area by 61%, boosts Pb^2+^ adsorption selectivity, and cuts charge-transfer resistance by 78%, linking DES-induced surface activation to metal coordination.

## Graphical abstract

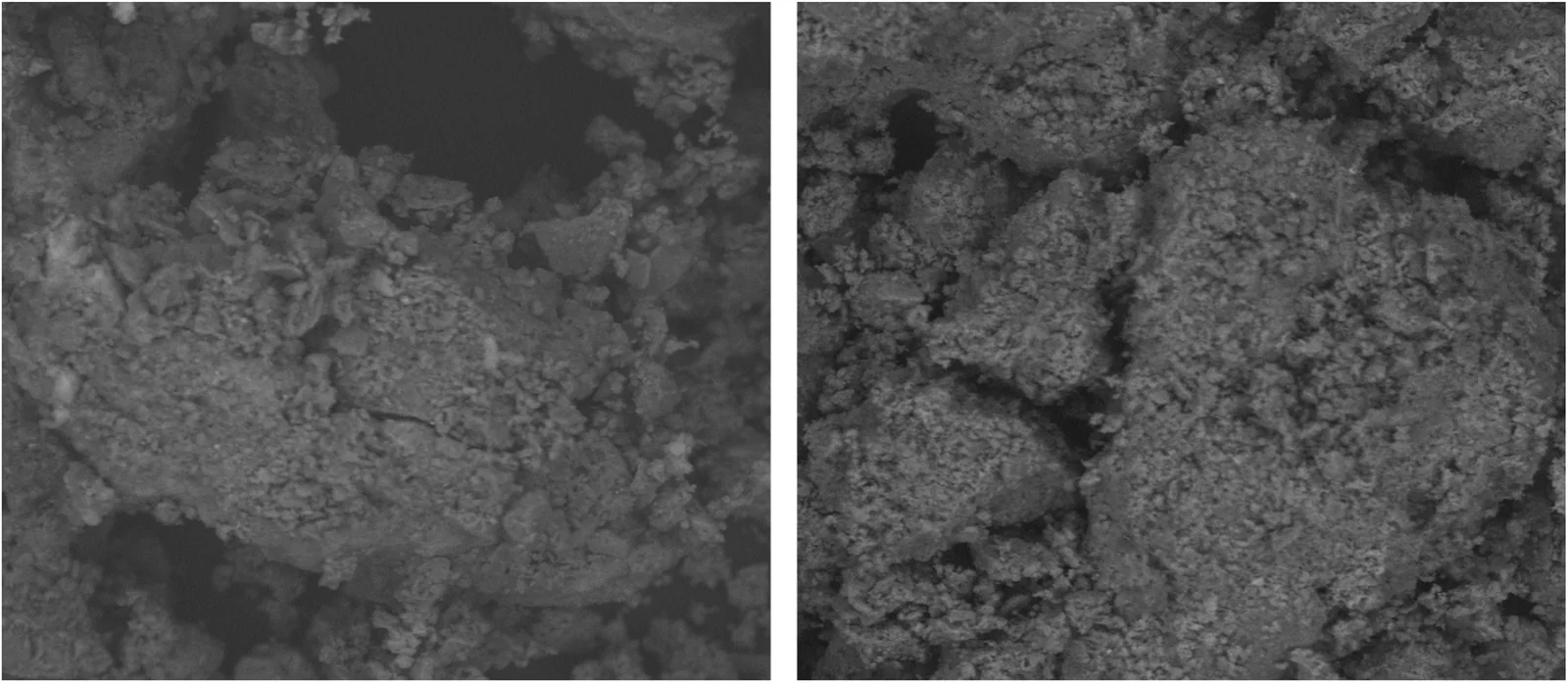

## 1 Introduction

The stabilization of dispersed metal species at solid interfaces is still a major issue in materials chemistry, especially in applications spanning heterogeneous catalysis, environmental remediation, and electrochemical energy conversion. The catalytic response of such systems is governed not only by the intrinsic activity of the metal centers, but also by the physicochemical attributes of the supporting matrix, which include surface functionality, structural coherence, and local coordination environments. These factors govern metal dispersion, anchoring strength, and the durability of the system under prolonged use. Carbonaceous supports and transition metal oxides have underpinned much of modern catalytic design, but their deployment is frequently constrained by corrosion susceptibility, limited chemical tunability, synthetic cost, or structural degradation under extended operation. Because of this, there is growing interest in simpler and more abundant materials that can offer both stability, compositional flexibility and environmental compatibility.^1^

Layered aluminosilicates, and montmorillonite in particular, are good candidates in this regard. They offer a structurally defined platform for interfacial engineering. Its 2 : 1 tetrahedral–octahedral–tetrahedral (TOT) architecture generates a permanent negative layer charge balanced by exchangeable interlayer cations, conferring a substantial cation exchange capacity and allowing incorporation of metal species within confined galleries. This interlayer chemistry provides a means of regulating metal dispersion and accessibility through ion exchange and surface coordination processes. However, pristine montmorillonite presents a relatively limited population of accessible coordination sites, and the inherent metal–support interactions are often insufficiently strong to stabilize active species under demanding catalytic or electrochemical conditions. As a result, unmodified clay rarely meets the requirements of advanced functional systems without prior surface or structural modification.^2^

In order to address this, different modification methods have been used over the years. These include acid treatment, pillaring with metal oxides, organic intercalation, and thermal processing. Such methods can open up the structure, increase surface area, or introduce new active sites. Pillared montmorillonite systems incorporating Al/Mn oxide pillars and polymeric components, for instance, have demonstrated enhanced surface area and strengthened chelation of Pd nanoparticles, resulting in improved activity in cross-coupling reactions.^3^ However, conventional treatments can introduce heterogeneity in site distribution, partial loss of crystallographic order, or even layer collapse. Such structural perturbations compromise mechanical integrity during subsequent metal loading and may deteriorate long-term performance.^2^ The difficulty therefore, is achieving meaningful interfacial reconfiguration while preserving the layered framework that confers montmorillonite its intrinsic hosting capability.

Deep eutectic solvents (DESs) are now being explored as an alternative medium for materials synthesis and surface modification. Their advantages include chemical versatility and environmental compatibility. Formed through complexation between a hydrogen bond acceptor, such as choline chloride, and a hydrogen bond donor, including urea or ethylene glycol, DESs show tunable polarity, viscosity, and coordination behaviour. These physicochemical attributes allow them to function simultaneously as solvents, structure-directing agents, and reactive participants. Their low vapor pressure, biodegradability, comparatively low toxicity, and economic accessibility align closely with green chemistry principles.^1,4^

Many studies have shown the capacity of DES media to modulate nucleation, crystal growth, defect chemistry, and heteroatom incorporation during the formation of inorganic and nanostructured materials.^5^ In such systems, the solvent environment actively shapes structural evolution, yielding materials with enhanced catalytic, adsorptive, or electrochemical properties. DES-assisted routes have, for example, facilitated the preparation of transition metal oxides subsequently integrated into polymer matrices for supercapacitor applications, where improvements in capacitance and operational stability were achieved relative to conventional synthesis pathways.^6^

In electrocatalysis, DESs have also served as templating media and reactive components in the fabrication of cobalt-based catalysts for water splitting, with the solvent constituents contributing to heteroatom incorporation and modification of active site environments.^7^ The development of metal salt-based DESs (MSDESs) further extends this concept by embedding metal salts directly into the eutectic framework, thereby generating solvent systems with intrinsic compositional functionality and potential activity in catalysis, gas absorption, and sorption processes.^8^ These observations highlight the capacity of DES environments to influence both composition and structure in a manner difficult to replicate using conventional solvents.

However, comparatively limited attention has been devoted toward the use of DESs for controlled engineering of layered clays into stable metal-hosting scaffolds. Existing studies predominantly focus on DES functionalization of carbonaceous adsorbents or the synthesis of porous frameworks, whereas systematic investigation of DES–montmorillonite interactions and their impact on metal coordination environments remains limited.^9^ Molecular-scale analyses have shown that montmorillonite edge sites exhibit dynamic protonation behaviour and pronounced pH-dependent reactivity, factors that directly influence ion exchange and interfacial coordination in chemically modified environments.^10^ This suggests that DES-mediated treatments may alter not only interlayer spacing but also the local chemistry governing metal anchoring.

A mechanistic understanding of how DES processing modifies clay structure, and how such modifications relate to the metal hosting capacity, has yet to be established. Without that connection, rational design of clay-based catalytic scaffolds that utilize both the structural order of layered silicates and the synthetic advantages of DES media remains constrained.

The present study addresses this gap through systematic investigation of a zinc chloride–urea metal salt-based DES as a surface modification agent for montmorillonite. Unlike prior work focused on DES functionalization of carbonaceous adsorbents or porous frameworks,^9^ this study targets layered aluminosilicate clay and uses multi-ion adsorption as a diagnostic probe of surface activation rather than as a primary remediation objective. The use of ZnCl_2_ as a hydrogen bond acceptor and reactive component simultaneously distinguishes this approach from choline chloride-based systems. Structural evolution is evaluated through XRD, FTIR, SEM, BET physisorption, and zeta potential measurements before and after DES treatment and metal loading, with electrochemical characterization included to assess the implications of surface reconfiguration for interfacial charge-transfer behaviour. By establishing the mechanistic link between DES-induced structural modification and metal coordination capacity, the work provides a basis for developing clay-based scaffolds for catalytic and electrochemical applications.

## 2 Experiment

### 2.1 Materials

Montmorillonite clay was sourced from a natural deposit in Ngala, Borno State. SEM-EDS analysis showed that the montmorillonite is predominantly composed of silicon (Si, 41.83 atomic%), aluminum (Al, 17.48 atomic%), and sodium (Na, 21.42 atomic%). Lower concentrations of iron (Fe, 5.95 atomic%), chlorine (Cl, 4.92 atomic%), magnesium (Mg, 2.54 atomic%), potassium (K, 1.89 atomic%), titanium (Ti, 1.10 atomic%), sulfur (S, 1.55 atomic%), and phosphorus (P, 1.32 atomic%) are also identified. The relatively high sodium content confirms that Na^+^ is the predominant exchangeable cation, supporting a sodium-rich montmorillonite composition. Zinc chloride (ZnCl_2_, >98% purity), urea (CO(NH_2_)_2_, >98% purity), and deionized water, were purchased from 5-Star Chemicals Limited. All reagents used were analytical grade. The initial concentrations of chromium (Cr), cadmium (Cd), and lead (Pb) in the water samples were determined using Varian SpectrAA Atomic Absorption Spectrometer (AA).

### 2.2 Preparation of deep eutectic solvent (DES)

The DES was prepared by combining zinc chloride and urea in a 1 : 2 molar ratio. The mixture was heated at 80 °C under continuous stirring until a homogeneous, colourless liquid was obtained. The resulting eutectic was used immediately for clay modification without further purification.

### 2.3 Surface modification of montmorillonite

Montmorillonite (5 g) was dispersed in 50 mL of the prepared DES and maintained at 80 °C under continuous stirring for 2 h. This treatment promotes interaction between the DES and the montmorillonite surface, leading to redistribution of surface hydroxyl groups and partial reorganization of interlayer interactions. The modified montmorillonite was thoroughly rinsed with deionized water to remove residual DES components and subsequently dried at 60 °C for 12 h. The dried material was subsequently ground to a fine powder to ensure uniformity in subsequent characterization and adsorption studies.

### 2.4 Characterization

The structural and chemical features of the DES-treated montmorillonite were characterized using X-ray diffraction (XRD), Fourier-transform infrared spectroscopy (FTIR), and scanning electron microscopy combined with energy-dispersive X-ray spectroscopy (SEM–EDS).

XRD measurements were conducted to evaluate whether the layered aluminosilicate framework was retained after DES treatment, with a focus on changes in basal spacing and overall crystallinity. FTIR analysis provided insight into modifications of surface functional groups, such as hydroxyl species and Si–O–Al vibrational modes associated with chemical activation. Surface morphology and textural features, including platelet arrangement and porosity were examined *via* SEM, while EDS analysis was performed to determine elemental composition and assess the distribution of exchangeable cations within the montmorillonite matrix.

#### 2.4.1 X-ray diffraction (XRD)

Diffraction patterns for the raw, DES-modified, and post-adsorption samples were collected using a Rigaku MiniFlex diffractometer operating in *θ*–2*θ* configuration. Measurements were conducted using Cu Kα radiation (*λ* = 1.5406 Å) generated from a copper-target X-ray tube. Data acquisition was carried out in step-scan mode using a step size of 0.02° in 2*θ*. All measurements were performed at room temperature under ambient atmospheric conditions.

#### 2.4.2 Fourier-transform infrared spectroscopy (FTIR)

Infrared spectra were recorded using an Agilent FTIR spectrometer. Measurements were conducted over the range 4000–650 cm^−1^ with a spectral resolution of 16 cm^−1^. For each spectrum, 64 scans were accumulated for both the background and the sample. Samples were prepared using the KBr pellet method, in which approximately 1 mg of finely ground material was thoroughly mixed with 100 mg of spectroscopic-grade KBr and pressed into a transparent pellet prior to analysis.

#### 2.4.3 Scanning electron microscopy and energy-dispersive X-ray spectroscopy (SEM-EDS)

Surface morphology and elemental composition were characterized using a scanning electron microscope equipped with a backscattered electron detector. Imaging was conducted at an accelerating voltage of 15 kV under high-vacuum conditions. Elemental analysis was performed using EDS in point analysis mode.

#### 2.4.4 Zeta potential measurements

Zeta potential measurements were conducted using a Malvern Zetasizer Nano ZS instrument. Samples were dispersed in 1 mM KNO_3_ background electrolyte at a concentration of 1 mg mL^−1^, and the solution pH was adjusted using 0.1 M HNO_3_ and 0.1 M KOH. Measurements were performed over the pH range 2–10 at 25 °C, with each reported value representing the mean of three independent measurements (*n* = 3). The point of zero charge (PZC) was determined by linear interpolation of the zeta potential–pH profile at the zero-crossing.

#### 2.4.5 BET surface area and pore structure

N_2_ physisorption measurements were conducted at 77 K using a Micromeritics ASAP 2020 analyser. Samples were degassed at 150 °C for 12 h under vacuum prior to analysis. BET surface area was determined using the BET method, while total pore volume, average pore diameter and pore size distributions were calculated using the BJH method.

#### 2.4.6 X-ray photoelectron spectroscopy (XPS)

X-ray photoelectron spectroscopy was performed using a monochromatic Al Kα X-ray source (1486.6 eV). A survey scan (0–1200 eV) was acquired at a pass energy of 160 eV to determine overall surface elemental composition (Na 1s, Zn 2p, O 1s, Si 2p, C 1s, Al 2p), with the adventitious C 1s peak referenced to 284.8 eV to correct for surface charging. High-resolution core-level scans were additionally acquired at a pass energy of 20 eV for Zn 2p and O 1s to resolve their chemical states. Peak fitting was carried out using a Shirley background with mixed Gaussian–Lorentzian line shapes.

### 2.5 Metal ion adsorption studies

Batch adsorption experiments were conducted in a 2 L batch reactor containing the selected contaminated water, with 50 mL aliquots withdrawn at predetermined intervals for AAS analysis. The initial concentrations of metal ions in the water samples, as determined by AAS prior to treatment, were as follows, Cr^3+^ at 0.452 mg L^−1^, Cd^2+^ at 0.128 mg L^−1^, and Pb^2+^ at 0.612 mg L^−1^. Adsorbent dosages of 0.5, 1.0, 1.5, 2.0, and 2.5 g were introduced into the solutions and stirred at 300 rpm at ambient temperature. The pH of all solutions was maintained at 7.0. Aliquots were withdrawn at predetermined intervals (20, 40, 60, 80, and 100 min), filtered, and analyzed to determine residual metal content using a Varian SpectrAA Atomic Absorption Spectrometer. All adsorption experiments were conducted in triplicate (*n* = 3), and results are reported as mean ± standard deviation (SD). Relative standard deviations (RSDs) for all reported adsorption capacities were below 5%, confirming reproducibility.

The equilibrium adsorption capacity was determined using:
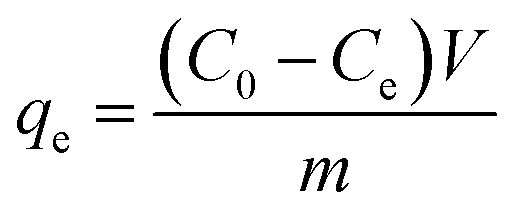
where *C*_0_ and *C*_e_ represent the initial and equilibrium metal concentrations (mg L^−1^), *V* is the solution volume (L), and *m* is the mass of adsorbent (g).

The effect of solution pH on adsorption capacity was examined over the range pH 2–10, with all other parameters fixed at their optimum values (adsorbent dosage: 1.5 g, and contact time: 80 min). Solution pH was controlled using 0.1 M HNO_3_ and 0.1 M NaOH.

Single-component adsorption isotherm experiments were conducted to determine Langmuir and Freundlich model parameters for each metal ion independently. Isotherm experiments were conducted over an initial concentration range of 0.10–5.00 mg L^−1^. For each concentration, 50 mL of single-metal solution was contacted with 1.5 g of DES-modified montmorillonite at pH 7.0 and 300 rpm for 80 min. Equilibrium concentrations were determined by AAS and adsorption capacities calculated using the equation above

The Langmuir model;
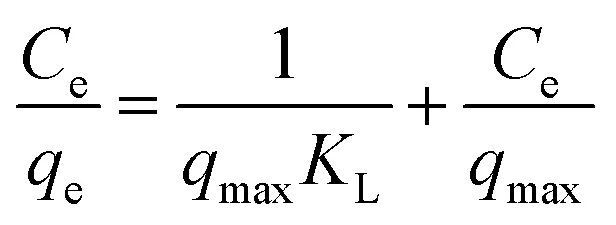
and Freundlich model;
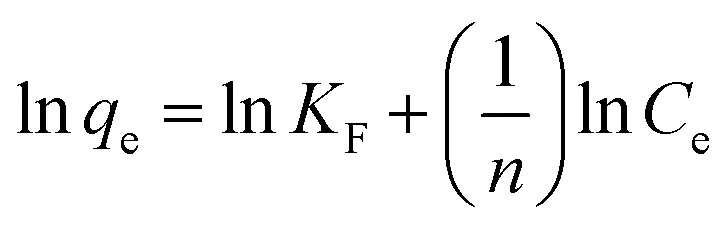
were applied in their linearised forms with parameters obtained by linear regression.

Recyclability experiments were conducted over five consecutive adsorption–regeneration cycles. Following each adsorption cycle, the spent adsorbent was regenerated by contact with 50 mL of 0.1 M HNO_3_ for 60 min at 300 rpm, followed by three deionised water washes (50 mL each) and drying at 60 °C for 12 h.

To provide a direct, condition-matched comparison, adsorption experiments were additionally performed using unmodified (natural) montmorillonite under identical conditions to those used for the DES-modified material, the same real contaminated water matrix, pH 7.0, 300 rpm, 80 min contact time, and adsorbent dosages of 0.5–2.5 g in a 2 L batch reactor.

### 2.6 Electrochemical characterization

Electrochemical measurements were carried out using a Biologic SP-200 potentiostat in a conventional three-electrode cell consisting of a glassy carbon electrode as the working electrode, a platinum wire as the counter electrode, and an Ag/AgCl (3 M KCl) electrode as the reference electrode. The supporting electrolyte consisted of 5 mM ferri/ferrocyanide solution containing 0.1 M KCl at pH 7.0.

Working electrodes were prepared by dispersing 3 mg of unmodified montmorillonite and DES-modified montmorillonite separately in 1 mL of deionised water under sonication for 15 min. A 5 µL aliquot of the resulting suspension was drop-cast onto the polished glassy carbon electrode (GCE) surface and allowed to dry at room temperature. Prior to modification, the GCE was polished sequentially using 1.0, 0.3 and 0.05 µm alumina slurry, followed by sonication in deionised water and ethanol.

Cyclic voltammetry (CV) measurements were recorded over the potential range −0.6 to +0.8 V *versus* Ag/AgCl at a scan rate of 50 mV s^−1^. Electrochemical impedance spectroscopy (EIS) measurements were conducted at open-circuit potential using a 10 mV sinusoidal perturbation over a frequency range of 10^5^ to 10^−2^ Hz. All CV and EIS measurements were performed in triplicate (*n* = 3) on independently prepared electrodes; values are reported as mean ± SD.

## 3 Results and discussion

### 3.1 DES-induced structural reconfiguration of montmorillonite

The XRD patterns obtained before and after treatment with the ZnCl_2_–urea deep eutectic solvent show that the fundamental layered structure of montmorillonite was retained throughout the study (Fig. 1). A broad, low-intensity reflection centered near 2*θ* = 19–21° is present in all three diffractograms (raw, DES-modified, and post-adsorption samples) and is characteristic of the (02,11) reflection band of montmorillonite, a poorly crystalline, turbostratically disordered mineral that typically produces broad rather than sharp basal reflections. A sharp, well-resolved reference peak in the 2*θ* at 26.5–27.0° region is present in all three samples. Database matching assigns this peak to quartz in the unmodified and DES-modified samples and to orthoclase in the post-adsorption sample, reflecting minor variation in the relative contribution of accessory feldspar and silica phases rather than a change in the montmorillonite framework itself. Additional reflections matched to quartz, albite, kaolinite, and chlorite phases across the three diffractograms confirm the mixed mineralogy typical of natural bentonite deposits.

**Fig. 1:**
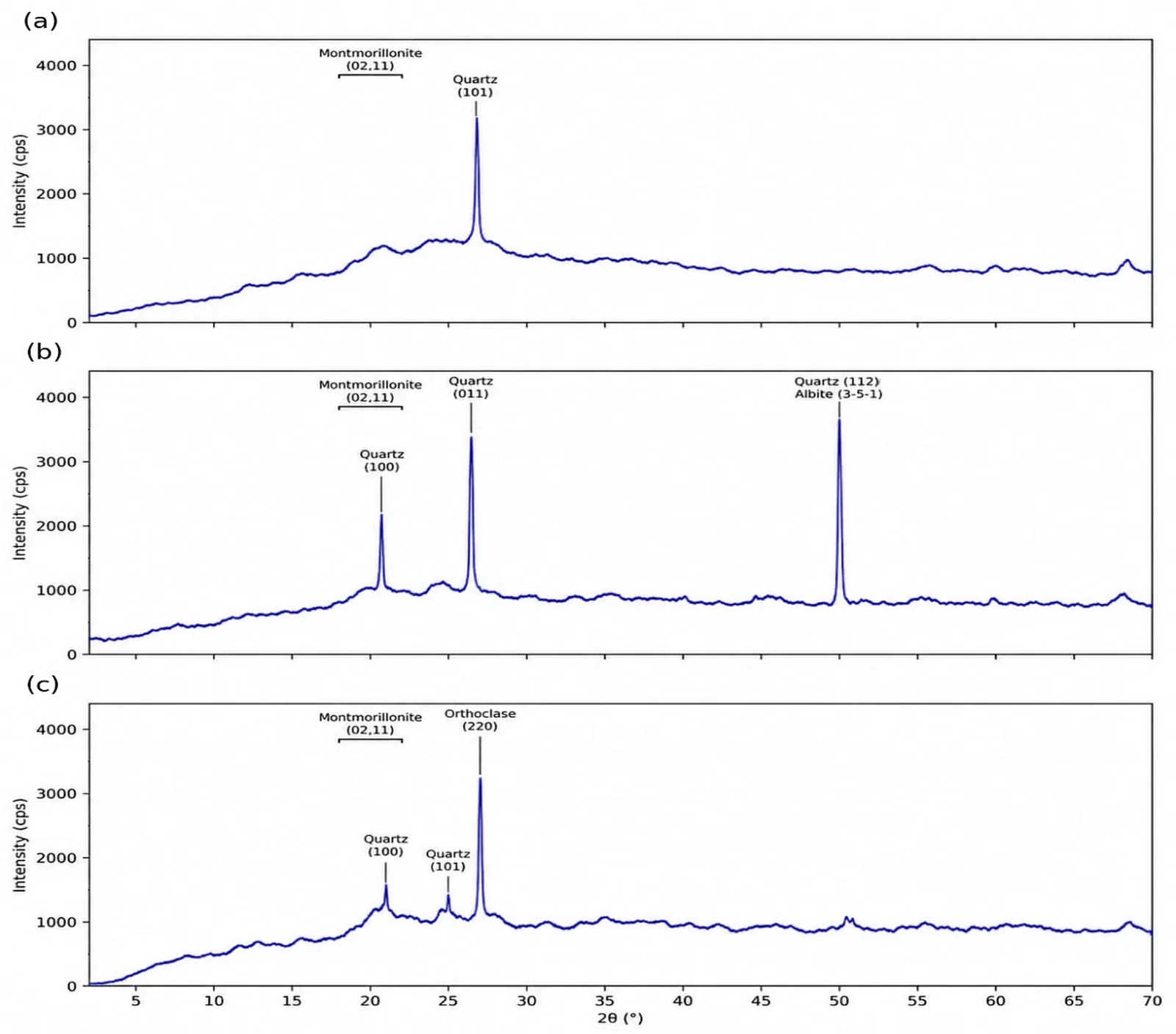
XRD patterns of, (a) unmodified, (b) DES-modified, and (c) post-adsorption montmorillonite.

The (001) basal reflection, expected in the low-angle region (2*θ* < 10°), could not be reliably resolved from the background in any of the three diffractograms at the count times used in this study (20° min^−1^ scan speed). Consequently, no quantitative interlayer *d*-spacing values are reported, and no conclusions regarding DES intercalation into the interlayer gallery are drawn from this reflection. This limitation is consistent with the generally weak, broad, and orientation-sensitive nature of clay basal reflections in natural, mixed-mineralogy samples measured under fast survey-scan conditions.

A reflection near 2*θ* at 20.7–21.0° is resolved as a discrete peak in the DES-modified and post-adsorption diffractograms, matched by database comparison to quartz (100), in the unmodified sample this region is dominated by the broad, unresolved montmorillonite hump, and no discrete peak is distinguishable above it. An additional sharp reflection near 2*θ* at 50.0°, matched to quartz (112)/albite, is prominent in the DES-modified sample but is not clearly resolved above background in either the unmodified or post-adsorption diffractograms. Because natural, mixed-mineralogy clays are heterogeneous at the sub-sample scale, differences in the resolution of minor accessory-mineral reflections across separately mounted aliquots are expected and do not, by themselves, indicate the appearance of a new crystalline phase. In every sample where these reflections are resolved, database matching identifies them as quartz or albite, with no phase specific to zinc identified at any stage. Evidence for zinc incorporation is instead provided independently by the EDS and XPS data presented in Sections 3.1.2 and 3.1.4.

For the post-adsorption sample (Fig. 1c), the broad montmorillonite reflection and the quartz/feldspar reference peak remain present at consistent positions, and no new peaks associated with metal hydroxide precipitation or framework degradation are detected, indicating that the layered structure remains stable during adsorption.

Evidence of chemical modification is further examined by Fourier-transform infrared (FTIR) spectroscopy (Fig. 2). The Si–O–Si stretching band at 998.9 cm^−1^ and the Si–O–Si bending band at 775.3 cm^−1^ retain their positions across the unmodified, DES-modified, and post-adsorption spectra, indicating that the aluminosilicate framework is structurally preserved throughout treatment and metal uptake. Both bands show a marked increase in relative transmittance after DES treatment, attributed to the removal of adsorbed impurities and partial exposure of the silicate surface. Following metal adsorption, transmittance at these bands decreases again, indicative of surface interaction between the exposed framework and adsorbed metal species, although FTIR intensity changes alone are not taken as quantitative evidence of this interaction. In the O–H stretching region, band narrowing and redistribution following DES treatment (Fig. 2b) reflect changes in hydrogen-bonding environments at accessible surface hydroxyl groups. Given the instrument's spectral resolution (16 cm^−1^), positional differences below this threshold are not interpreted as significant.

**Fig. 2:**
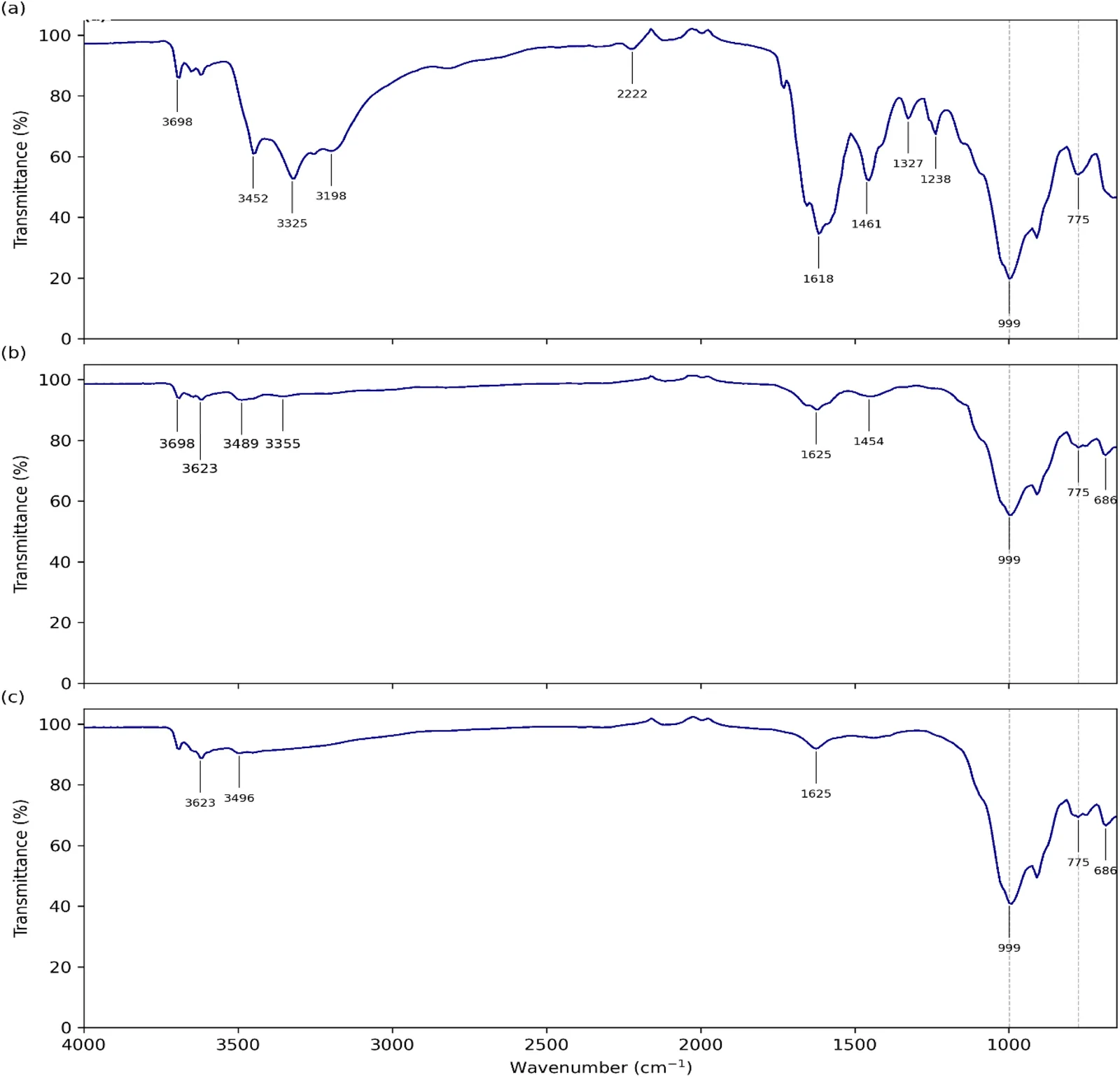
FTIR spectra of montmorillonite at (a) unmodified, (b) DES-modified, and (c) post-adsorption stages.

#### 3.1.1 Surface charge analysis

Zeta potential measurements were conducted to assess how DES treatment alters the surface charge of montmorillonite (Fig. 3). Unmodified montmorillonite shows a PZC near pH 2.5 and a zeta potential of −28.6 ± 1.6 mV at pH 7, arising from the permanent negative layer charge characteristic of Na-montmorillonite. Zeta potentials remain negative or near-zero across the full pH range. This demonstrates that structural charge dominates over variable edge contributions. Following DES modification, the PZC shifts to pH 3.2 and the zeta potential decreases in magnitude to −23.4 ± 1.5 mV at pH 7. This attenuation is attributed to partial charge screening by residual Zn^2+^ and Cl^−^ species retained at the surface, in agreement with the EDS and XPS data (Section 3.1.4) and additional XRD reflections discussed above. Post-adsorption montmorillonite shows the most negative zeta potential (−32.1 ± 1.7 mV at pH 7) and a PZC near pH 2.1 attributable to coordination of hydrolysed metal species at surface hydroxyl sites. The negative surface charge is maintained across the entire working pH range.

**Fig. 3:**
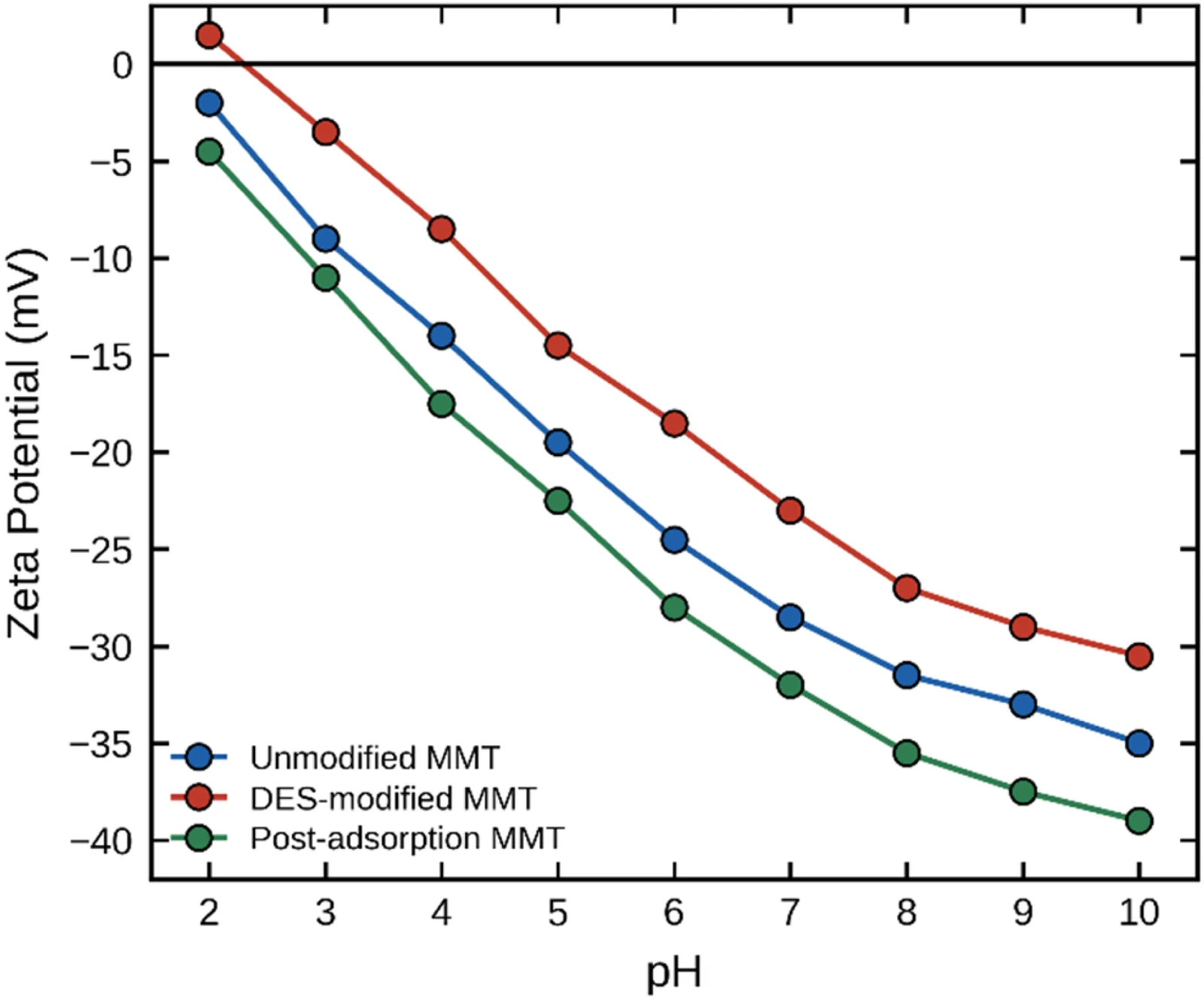
Zeta potential as a function of pH.

It should be noted that the PZC values reported here are electrokinetic in nature, derived directly from zeta potential as a function of pH, and therefore reflect the whole-particle surface response rather than the behaviour of edge sites in isolation. For 2 : 1 phyllosilicates such as montmorillonite, this response is dominated by the permanent negative structural charge from isomorphic substitution, which is pH-independent and persists across the measurable pH range, electrokinetically-derived PZC/IEP values for montmorillonite are consequently reported at low pH, generally below 3.^11^ This is distinct from potentiometric or mass-titration PZC, which isolates edge hydroxyl protonation/deprotonation while excluding the permanent basal charge, and is typically reported at pH 3.4–6.5 for montmorillonite.^12,13^

A further consideration is that the montmorillonite used in this study, sourced from Ngala, Borno State, is confirmed by EDS to be Na-rich, whereas Baba *et al.*, (2026) report that montmorillonite/bentonite from the Chad Basin, the broader geological region encompassing this deposit, is predominantly Ca-montmorillonite.^14^ The exchangeable cation population strongly influences surface charge and swelling behaviour, and this distinction, together with the ZnCl_2_–urea DES modification applied here, provides a material basis for the observed PZC values that is independent of measurement technique alone. The moderate PZC increase from pH 2.5 (unmodified) to 3.2 (DES-modified) is attributed to residual Zn^2+^/Cl^−^ retention at the surface, while the decrease to pH 2.1 after adsorption reflects occupation of surface hydroxyl sites by adsorbed metal species. The comparatively low absolute PZC values obtained here therefore reflect the electrokinetic measurement approach, exchangeable-cation chemistry, and DES-specific modification, rather than an anomaly in the material.

#### 3.1.2 Scanning electron microscopy

Scanning electron microscopy (SEM) was used to examine morphological changes across the three stages of montmorillonite treatment (Fig. 4). The unmodified montmorillonite (Fig. 4a and b) displays aggregated, blocky particles with relatively smooth surfaces and well-defined interparticle voids.

**Fig. 4:**
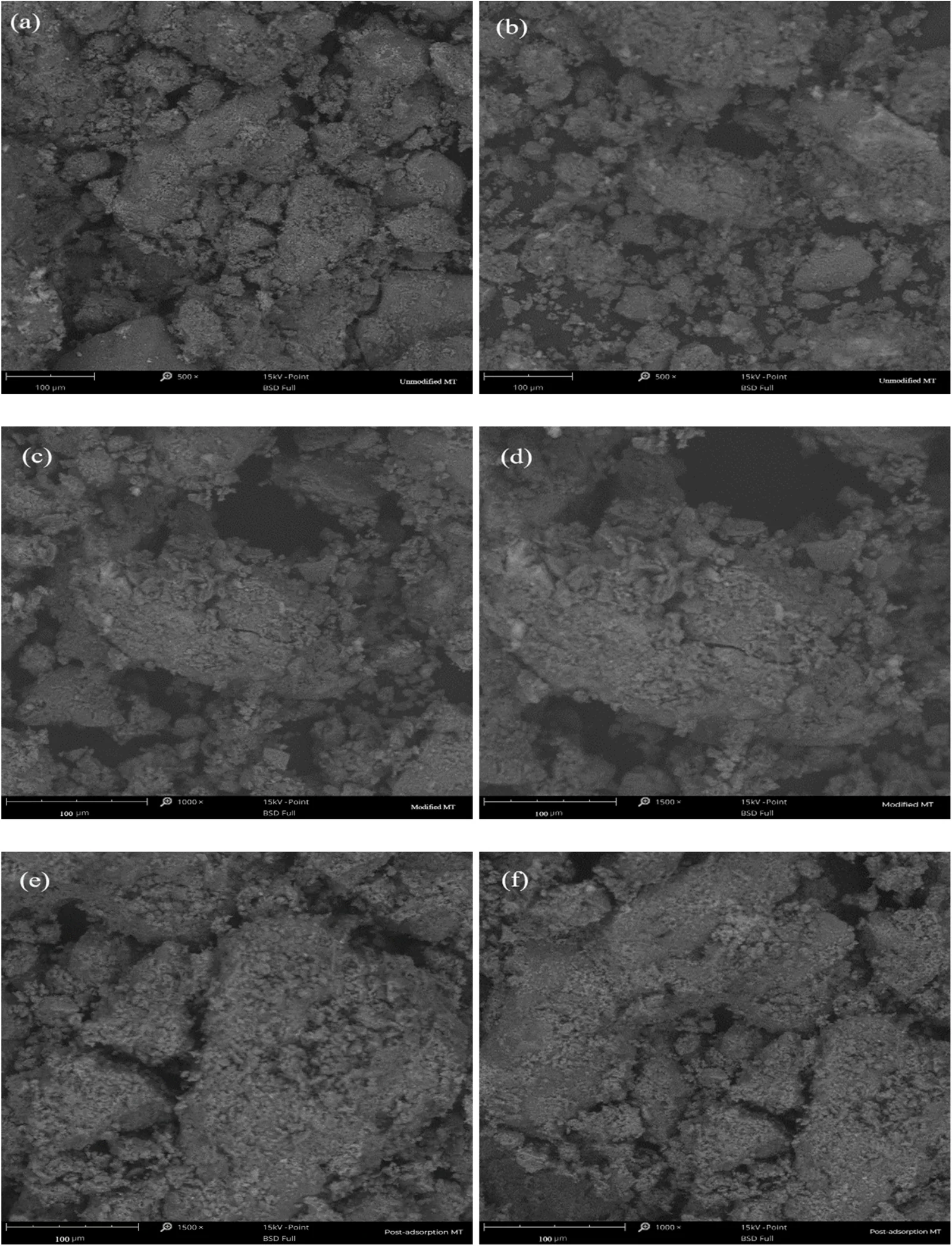
SEM images of (a and b) DES-unmodified montmorillonite, (c and d) modified montmorillonite, and (e and f) post-adsorption montmorillonite.

Following DES modification (Fig. 4c and d), a clear change in surface texture is observed. The particle surfaces appear noticeably rougher, with greater fragmentation of aggregate boundaries and increased exposure of platelet edges. These morphological changes are consistent with the redistribution of interlayer cations and partial incorporation of DES components into the gallery space, as indicated by the XRD and FTIR results. The increased edge exposure is consistent with the redistribution of interlayer cations and partial DES incorporation indicated by the XRD and FTIR results.

Post-adsorption micrographs (Fig. 4e and f) show a finer, more uniformly distributed particulate layer across the aggregate surfaces, consistent with occupation of surface sites by Cr^3+^, Cd^2+^, and Pb^2+^ ions. The overall aggregate architecture remains intact, confirming that neither DES treatment nor subsequent metal uptake causes structural collapse of the layered framework. EDS analysis confirms that the montmorillonite retains its primary aluminosilicate composition dominated by Si, Al, and Na across all three stages.

#### 3.1.3 Surface area and pore structure

N_2_ physisorption at 77 K was used to quantify changes in surface area and pore structure arising from DES treatment (Fig. 5). Unmodified montmorillonite exhibits a BET surface area of 42.3 ± 1.8 m^2^ g^−1^ and a total pore volume of 0.048 ± 0.003 cm^3^ g^−1^ as expected for the dense platelet arrangement of natural Na-montmorillonite. Following DES modification, the surface area increases to 68.1 ± 2.4 m^2^ g^−1^ (61% enhancement) and the total pore volume to 0.082 ± 0.004 cm^3^ g^−1^ (71% increase). These changes align with the SEM evidence of increased surface roughness and expanded platelet edge exposure, and confirm that DES treatment generates new accessible surface area without disrupting the primary lamellar structure, as evidenced by XRD. Post-adsorption montmorillonite shows a reduction in surface area and pore volume, consistent with partial occupation of surface sites by adsorbed metal species. Mean pore diameters remain relatively constant across all samples, increasing slightly from 4.52 ± 0.18 nm for unmodified montmorillonite to 4.81 ± 0.22 nm following DES treatment, before decreasing to 4.63 ± 0.19 nm after adsorption. All samples exhibit Type IV isotherms with H3 hysteresis, characteristic of slit-like mesopores associated with platelet stacking.

**Fig. 5:**
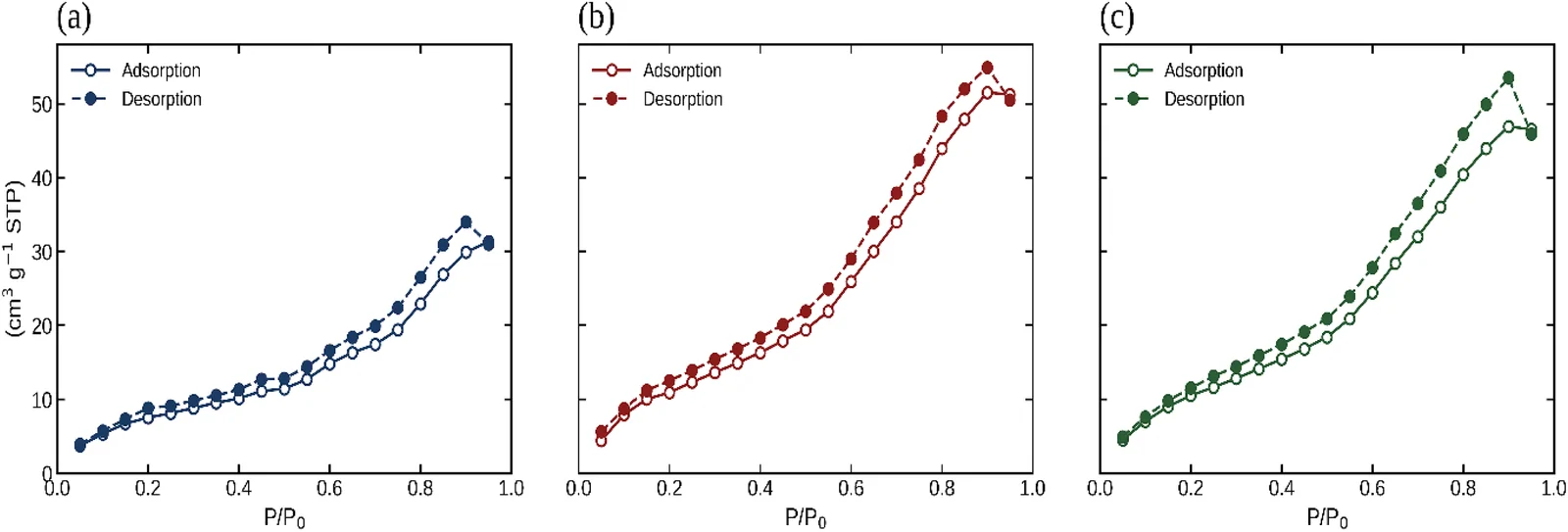
N_2_ adsorption–desorption isotherms of (a) unmodified, (b) DES-modified, and (c) post-adsorption montmorillonite.

#### 3.1.4 X-ray photoelectron spectroscopy (XPS)

To directly address the extent of zinc retention on the modified clay surface, and to complement the semi-quantitative, point-based elemental information provided by EDS, X-ray photoelectron spectroscopy (XPS) was performed on the DES-modified montmorillonite. Unlike EDS, which probes discrete micron-scale points selected during SEM imaging, XPS is a surface-averaged, self-calibrating technique (typical sampling depth of 5–10 nm) that yields a quantitative atomic composition independent of any single imaging field, providing an independent and more rigorous basis for assessing zinc incorporation.

The survey spectrum (Fig. 6a) confirms the expected clay framework elements (O 1s, Si 2p, Al 2p) together with Na 1s from the native exchangeable cation population and C 1s from adventitious carbon and residual DES-derived (urea) species. A distinct Zn 2p signal is resolved at approximately 1022 eV, together with the corresponding Zn 3s and Zn 3p transitions at lower binding energy, confirming that zinc from the ZnCl_2_–urea DES is retained on the clay surface after washing. This same survey scan, acquired on unmodified montmorillonite as a control (Table 1), shows no detectable Zn signal, establishing that the observed zinc originates from DES treatment rather than the parent clay. No chloride signal (Cl 2p, 198–200 eV; Cl 2s, ∼270 eV) was detected in the survey spectrum, indicating that unreacted DES and associated chloride counter-ions were effectively removed during washing and supporting incorporation of zinc as a surface-bound species rather than as residual physisorbed ZnCl_2_.

**Fig. 6:**
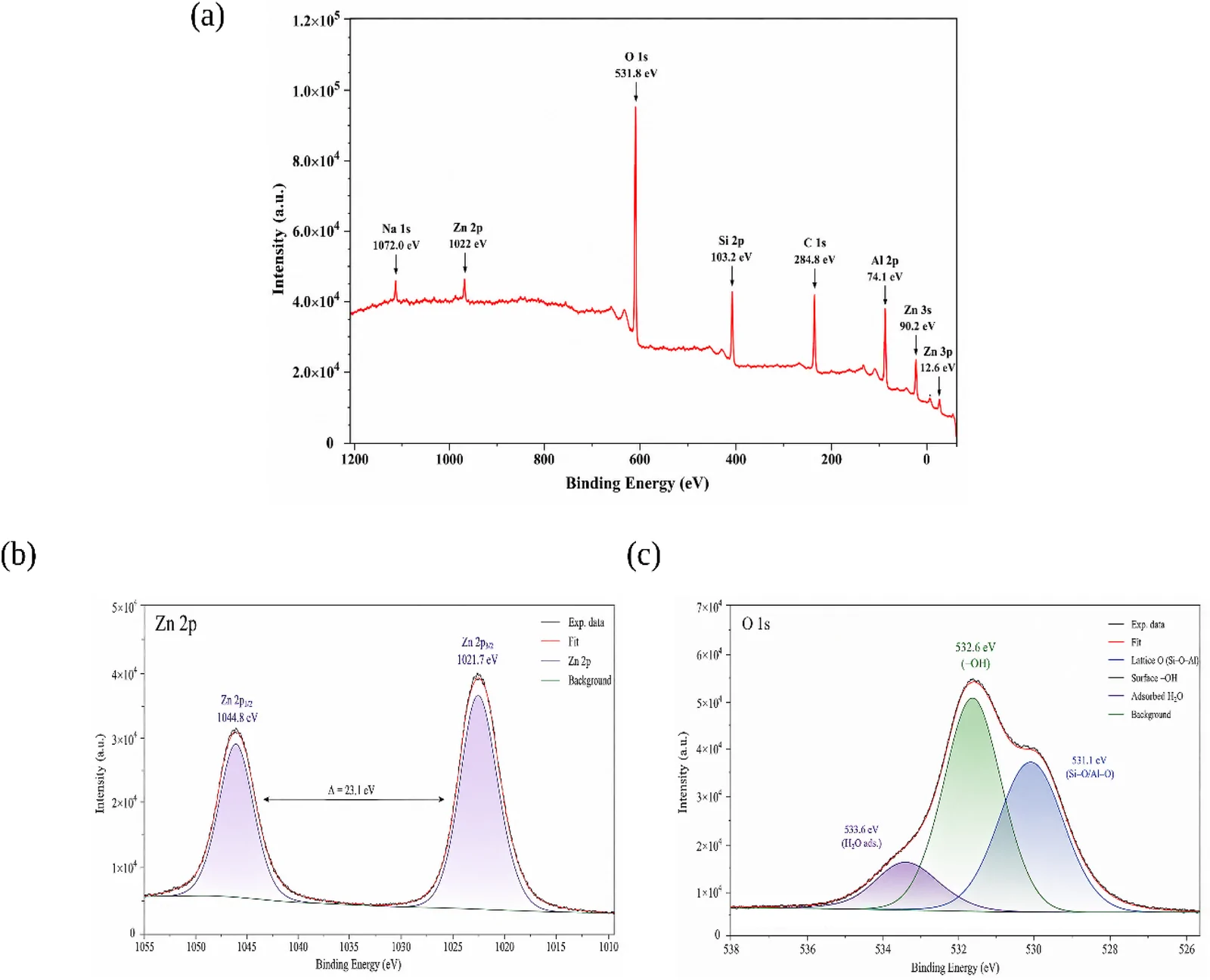
X-ray photoelectron spectroscopy of DES-modified montmorillonite, (a) survey spectrum, (b) high-resolution Zn 2p spectrum, and (c) deconvoluted O 1s.

**Table 1:** XPS-derived surface atomic concentrations of unmodified and DES-modified montmorillonite

Element	Natural MMT (atomic%)	DES-modified MMT (atomic%)
O 1s	65.5	63.9
Si 2p	20.4	19.6
Al 2p	7.6	7.1
Na 1s	2.6	1.9
Zn 2p	Not detected	0.74
C 1s	3.9	6.7

High-resolution Zn 2p spectra (Fig. 6b) resolve a well-defined spin–orbit doublet at 1021.7 eV (Zn 2p_3/2_) and 1044.8 eV (Zn 2p_1/2_), with a splitting of *Δ* = 23.1 eV, in close agreement with the accepted value for Zn^2+^ environments. Because the Zn 2p_3/2_ binding energies of metallic Zn^0^ (1021.8 eV) and Zn^2+^ oxide/hydroxide species (1021.9–1022.0 eV) are known to overlap closely, binding energy alone cannot unambiguously discriminate oxidation state. However, the aqueous, non-reducing conditions used throughout DES synthesis and washing provide no chemical pathway for Zn^2+^ reduction to Zn^0^, and the assignment to surface-coordinated Zn^2+^ (Zn–O–Si and/or Zn–OH) is further supported by the concurrent growth of the hydroxyl-associated O 1s component discussed below, together with the FTIR and zeta-potential evidence for increased surface hydroxylation (Sections 3.1.1 and 3.1.2).

Deconvolution of the O 1s envelope (Fig. 6c) resolves three components, a dominant surface hydroxyl contribution at 532.6 eV (–OH, encompassing Si–OH, Al–OH, and, consistent with the Zn 2p assignment above, Zn–OH), a lattice oxygen contribution at 531.1 eV (Si–O/Al–O), and a minor contribution at 533.6 eV attributed to adsorbed molecular water. The hydroxyl component being the dominant contribution to the O 1s envelope, rather than the lattice oxygen signal, corroborates the FTIR-based interpretation (Section 3.1.1) that DES treatment substantially redistributes and increases accessible surface hydroxyl groups rather than altering the aluminosilicate framework itself, and is consistent with the increase in point of zero charge from pH 2.5 to 3.2 attributed to residual Zn^2+^ retention (Section 3.1.2).

Quantitative atomic concentrations obtained from high-resolution XPS scans (Table 1) show zinc at 0.74 atomic% on the DES-modified surface, with no zinc detected on the untreated clay under identical acquisition conditions. This provides direct, quantitative confirmation of zinc incorporation, addressing the referee's request for zinc quantification independent of the EDS point analyses reported in Section 3.1.3. The XPS data further show a modest decrease in Na 1s concentration (2.6 to 1.9 atomic %) alongside the emergence of the Zn 2p signal, consistent with partial exchange of interlayer/surface Na^+^ for Zn-bearing species during DES treatment, and an increase in C 1s concentration (3.9 to 6.7 atomic%) consistent with retained urea-derived carbon from the DES. These results establish that zinc is retained on the modified clay surface predominantly as a surface-coordinated Zn^2+^ species (Zn–O–Si/Zn–OH), at a quantified concentration of 0.74 atomic%, without measurable disruption of the Si, Al, or framework O signals, consistent with the surface-level rather than structural modification described throughout Section 3.1.

### 3.2 Adsorption performance and metal–binding behaviour

The metal-hosting capacity of the DES-modified montmorillonite was evaluated using chromium (Cr^3+^), cadmium (Cd^2+^), and lead (Pb^2+^) ions as model systems. These metals differ in their ionic properties and coordination behaviour, making them suitable for examining how surface chemistry influences adsorption. Metal uptake depended on both the adsorbent dosage and contact time, a reflection of the balance between available binding sites, ion affinity, and competition within the system.

#### 3.2.1 Effect of adsorbent dosage

Adsorption capacities obtained at different adsorbent loadings are summarized in Table S13. Chromium adsorption shows marginal variation across the dosage range examined, increasing slightly from 0.0190 mg g^−1^ at 0.5 g to 0.0204 mg g^−1^ at 1.5 g before decreasing marginally at higher dosages. This is attributable to gradual saturation of available adsorption sites and a declining metal-to-site ratio at higher dosages.

Cadmium follows a different trend, with the highest adsorption capacities occurring at lower adsorbent dosages. This trend suggests preferential occupation of high-energy sites at low dosage, followed by reduced incremental uptake at higher adsorbent loadings. This behaviour is further governed by competitive interactions among coexisting metal ions. The ordering of Cr^3+^ and Cd^2+^ varied marginally across dosage conditions owing to competitive ion dynamics. The overall selectivity hierarchy of Pb^2+^ > Cr^3+^ > Cd^2+^ reflects the equilibrium behaviour established under optimised contact time and pH conditions.

Lead exhibits a continuous increase in adsorption capacity with increasing adsorbent dosage. The adsorption capacity rises steadily from 0.293 mg g^−1^ at 0.5 g to 0.479 mg g^−1^ at 2.5 g. The observed enhancement can be attributed to strong affinity between Pb^2+^ ions and the activated surface of the modified montmorillonite. This behaviour follows the known tendency of lead ions to form stable surface complexes with hydroxyl-bearing mineral surfaces. Adsorption capacities were evaluated under near-ambient, multi-ion conditions representative of real contaminated water, and are therefore not directly comparable with single-component isotherm data reported at elevated concentrations.^15^ However, the present experiments were conducted using a real contaminated water matrix at near-ambient metal concentrations in a competitive multi-ion system, which inherently suppresses individual adsorption capacities relative to idealized single-component tests.

#### 3.2.2 Time-dependent adsorption behaviour

The evolution of adsorption capacity with contact time was examined using the optimal dosage determined above (1.5 g). Time-resolved contact experiments at higher sampling density (13 points, 5–120 min, Table S4) confirmed the trends observed previously, Pb^2+^ uptake increased steadily before stabilising after ∼80 min, Cr^3+^ uptake rose to a maximum near 60 min before declining, and Cd^2+^ exhibited rapid initial uptake within the first 5–20 min followed by a sustained decline. This pattern is consistent with a competitive adsorption process in which the higher-affinity Pb^2+^ progressively displaces the more weakly bound Cr^3+^ and Cd^2+^ from shared surface sites over the course of the experiment. Chromium adsorption increases gradually during the initial stages of the experiment, rising from 0.006 mg g^−1^ at 5 min to a maximum of 0.088 mg g^−1^ at 60 min. Beyond this point, the capacity decreases, reaching 0.034 mg g^−1^ at 100 min. The initial increase is consistent with progressive occupation of accessible coordination sites, whereas the subsequent decline arises from redistribution of adsorbed ions and competitive displacement processes within the mixed-metal system.

Cadmium adsorption is highest within the first 5 min of contact (0.058 mg g^−1^), the earliest point sampled, after which capacity declines steadily through the early and intermediate time intervals before stabilising at lower values between 0.013 and 0.020 mg g^−1^ for the remainder of the experiment. This pattern indicates that Cd^2+^ binding is fast but weak, with initially bound Cd^2+^ progressively displaced as adsorption equilibria evolve.

Lead adsorption follows a more sustained trajectory. The capacity increases from 0.163 mg g^−1^ at 5 min to a peak of 0.409 mg g^−1^ at 70 min, remains near this level through 80 min (0.399 mg g^−1^), and then declines gradually at longer contact times. Compared with chromium and cadmium, the more persistent uptake of Pb^2+^ highlights its stronger interaction with the activated surface sites generated by DES treatment.

### 3.3 Kinetic modelling

Kinetic data were fitted using non-linear least-squares regression of the original rate equations, rather than the linearized transformations used in the previous version of this manuscript, which are known to introduce systematic bias into the fitted parameters and goodness-of-fit statistics. Pseudo-first-order (PFO) and pseudo-second-order (PSO) models were applied to each metal, alongside a two-term exponential model *q*(*t*) = *A*(e^−*k*_dis_*t*^ − e^−*k*_up_*t*^) capable of describing concurrent uptake and displacement, consistent with the use of exponential kinetic formulations as alternatives to classical PFO/PSO models for adsorption at the solid/solution interface.^16^

Model selection was based on the Akaike Information Criterion (AIC) and parameter identifiability, rather than *R*^2^ alone, since goodness-of-fit is not a sufficient criterion when comparing models of differing complexity, an over-parameterised model can achieve a higher *R*^2^ by fitting noise rather than by describing a genuine physical process. Pb^2+^ uptake was best described by the PSO model (*q*_e_ = 0.373 ± 0.026 mg g^−1^, *k*_2_ = 0.365 ± 0.157 g mg^−1^ min^−1^, *R*^2^ = 0.65), consistent with a chemisorption-dominated process at its preferred binding sites. Cr^3+^ was best described by the PFO model (*q*_e_ = 0.057 ± 0.011 mg g^−1^, *k*_1_ = 0.041 ± 0.026 min^−1^, *R*^2^ = 0.50), the two-term exponential model returned a nominally higher *R*^2^ (0.64) but yielded statistically unidentifiable rate constants (relative standard errors exceeding 10^4^%), and was therefore rejected in favour of the simpler, properly constrained PFO description. Cd^2+^ exhibited the most pronounced non-monotonic behaviour, reaching maximum uptake within the first sampling interval and declining progressively thereafter. This was well described by the two-term exponential model (*A* = 0.062 ± 0.006 mg g^−1^, *k*_dis_ = 0.0198 ± 0.0036 min^−1^, *R*^2^ = 0.81), with both goodness-of-fit and parameter precision supporting its use over the classical monotonic models.

These results indicate that Pb^2+^, Cr^3+^, and Cd^2+^ do not share a common rate-determining mechanism in this mixed-metal system (Fig. 7). Pb^2+^ approaches equilibrium *via* classical second-order surface binding, while Cr^3+^ and Cd^2+^ are progressively displaced from initially occupied sites as Pb^2+^ accumulates, consistent with the competitive adsorption behaviour discussed in Section 3.2.2.

**Fig. 7:**
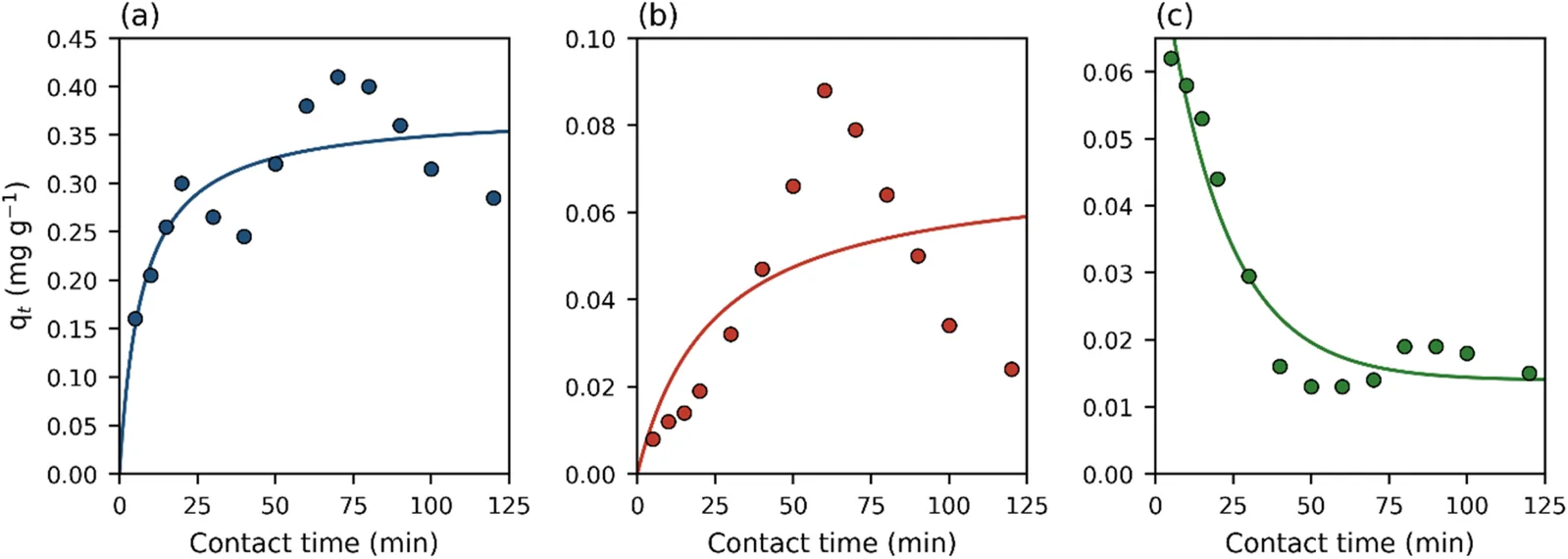
Non-linear kinetic fits of, (a) Pb^2+^ pseudo-second-order, (b) Cr^3+^ pseudo-first-order, and (c) Cd^2+^ two-term exponential uptake–displacement model.

The non-monotonic kinetic profiles observed for Cr^3+^ and Cd^2+^, an initial rise in adsorption capacity followed by partial decline at longer contact times reflect competitive displacement within the mixed-metal system. As Pb^2+^ accumulates preferentially on the highest-affinity hydroxyl sites, previously bound Cr^3+^ and Cd^2+^ are partially released, resulting in the observed decrease at 80–100 min. This behaviour is consistent with the selectivity hierarchy established by the isotherm *K*_L_ values and with competitive adsorption dynamics reported for mixed heavy metal systems on montmorillonite supports,^17,18^ the latter directly demonstrating displacement of pre-adsorbed Pb^2+^ and Co^2+^ from montmorillonite surfaces upon introduction of a competing cation. The more sustained adsorption trajectory of Pb^2+^ stems from its stronger surface complexation affinity, as further supported by its higher *K*_L_ (3.905 L mg^−1^) and lower 1/*n* (0.231) values relative to Cr^3+^ and Cd^2+^.

### 3.4 Effect of solution pH

The adsorption capacity of DES-modified montmorillonite shows a pronounced dependence on solution pH for all three metal ions. Adsorption is suppressed at low pH,^2–4^ where protonation of surface hydroxyl groups reduces the negative surface charge, and H^+^ competition limits metal access to coordination sites. Adsorption capacity increases with increasing pH, reaching metal-specific maxima before declining at higher pH (Fig. 8).

**Fig. 8:**
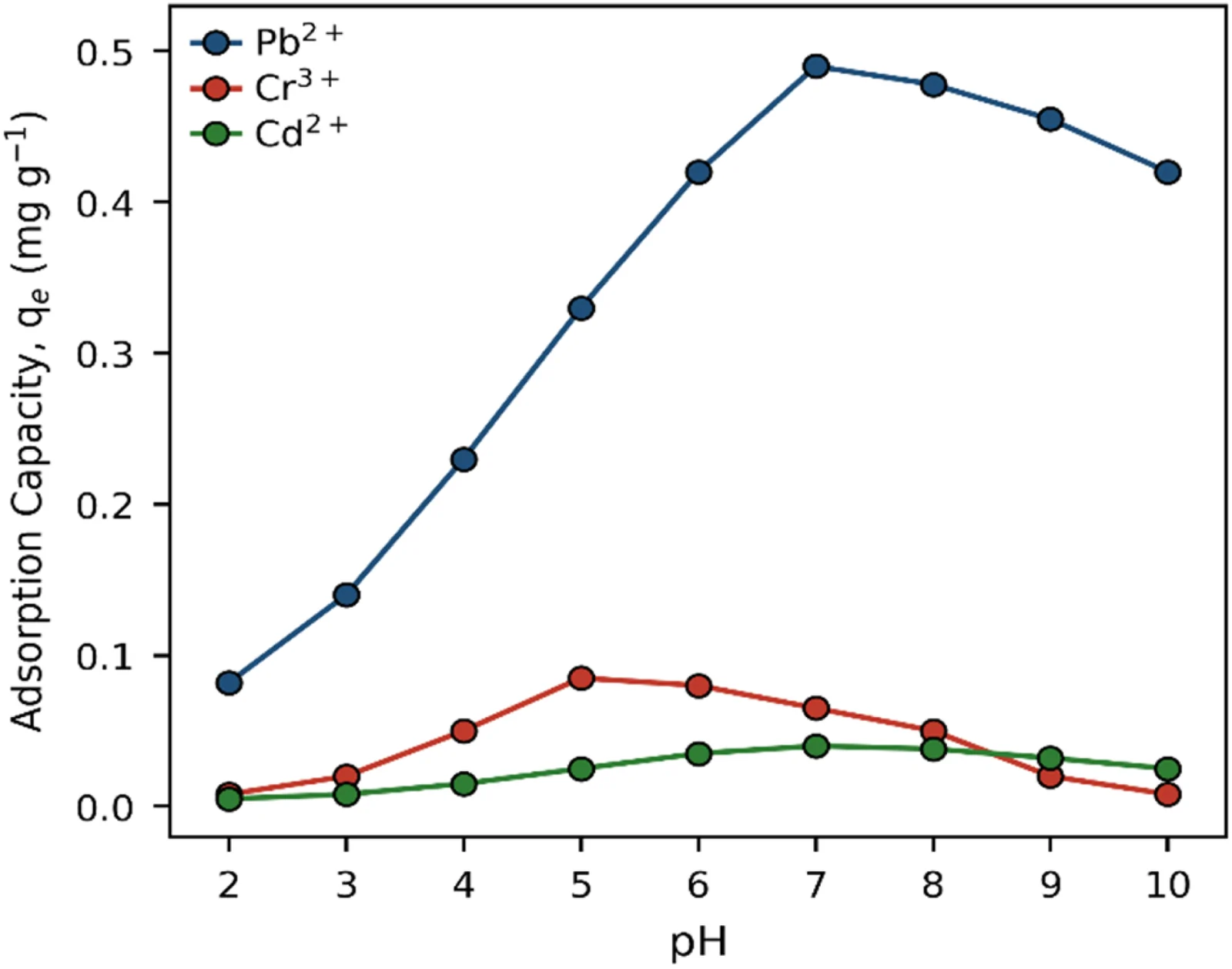
Effect of pH on adsorption capacity (*q*_e_) for Pb^2+^, Cr^3+^ and Cd^2+^.

Lead shows a broad adsorption plateau across pH 6–8, with a maximum of 0.479 mg g^−1^ at pH 7, confirming that pH 7 is a well-justified operational condition. Chromium peaks sharply at pH 5 (0.086 mg g^−1^) before declining at higher pH values, a behaviour attributable to progressive hydrolysis of Cr^3+^ and the onset of Cr(OH)_3_ precipitation, which reduces the concentration of freely available chromium species in solution. Cadmium shows a gentler pH dependence, peaking near pH 6–7 (0.051 mg g^−1^), a consequence of its comparatively weaker affinity for hydroxyl-mediated surface coordination. The selectivity order Pb^2+^ > Cr^3+^ > Cd^2+^ is maintained across pH 4–8 in agreement with the principal adsorption findings, supporting the conclusion that DES-activated surface hydroxyl groups govern metal uptake. pH 7.0 represents a practical compromise where adsorption capacities are near maximum for all three metals (Fig. 8).

### 3.5 Adsorption isotherms

Single-component isotherm experiments were conducted to determine maximum adsorption capacities free from competitive ion effects (Fig. 9). The Langmuir model provided the best fit for all three metals (Table 2), with *R*^2^ values of 0.989 (Pb^2+^), 0.993 (Cr^3+^), and 0.997 (Cd^2+^), a monolayer adsorption at energetically homogeneous surface sites. This is in accord with the chemisorptive pathway established by the kinetic modelling. The Freundlich model yielded lower *R*^2^ values (0.880–0.918) and 1/*n* values well below unity for all three metals, confirming favourable adsorption on a heterogeneously activated surface.

**Fig. 9:**
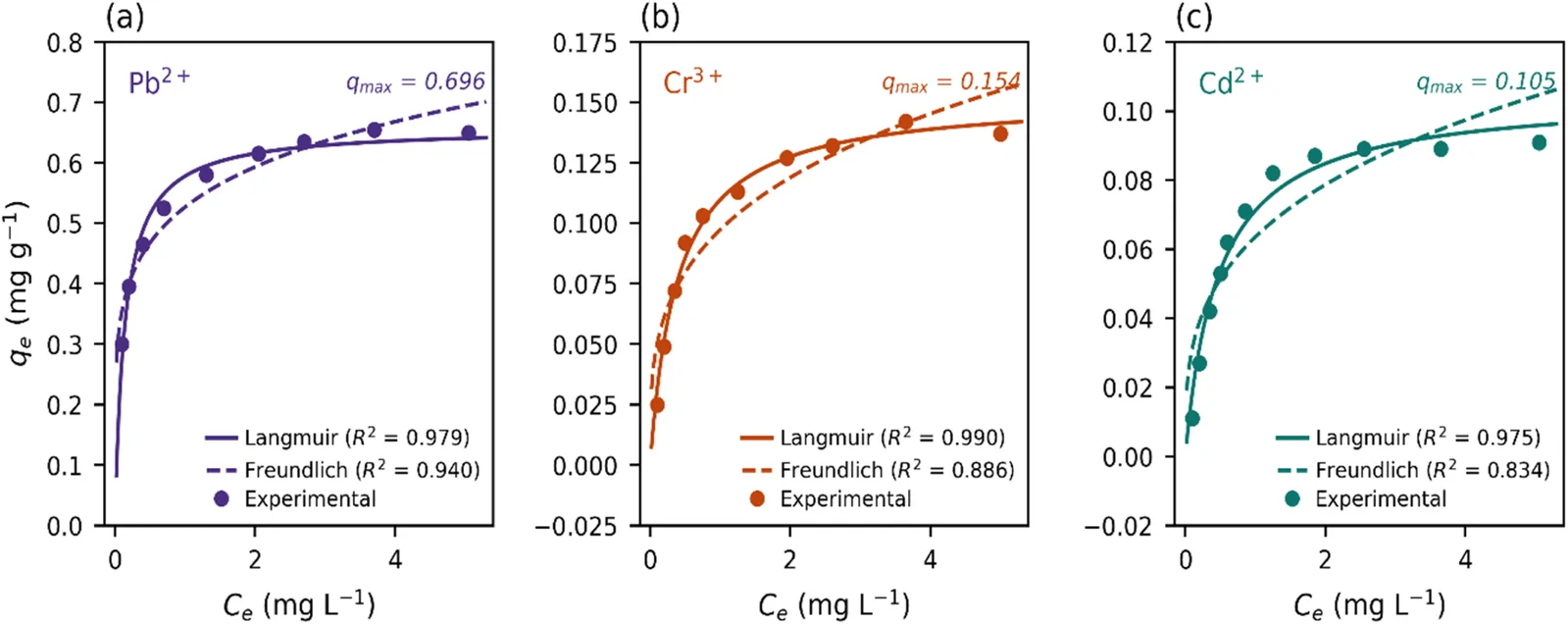
Single-component adsorption isotherms of, (a) Pb^2+^, (b) Cr^3+^, and (c) Cd^2+^.

**Table 2:** : Langmuir and Freundlich isotherm parameters for single-component adsorption of Pb^2+^, Cr^3+^, and Cd^2+^ on DES-modified montmorillonite

Metal	Langmuir			Freundlich		
	*q*_max_ (mg g^−1^)	*K*_L_ (L mg^−1^)	*R* ^2^	*K*_F_ (mg g^−1^)	1/*n*	*R* ^2^
Pb^2+^	0.696	3.905	0.989	0.502	0.231	0.880
Cr^3+^	0.154	2.283	0.993	0.096	0.299	0.906
Cd^2+^	0.105	1.702	0.997	0.060	0.341	0.918

Theoretical maximum adsorption capacities (*q*_max_) follow the order Pb^2+^ (0.696 mg g^−1^) > Cr^3+^ (0.154 mg g^−1^) > Cd^2+^ (0.105 mg g^−1^), consistent with the selectivity observed in the mixed-metal experiments. The Langmuir constant *K*_L_ follows the same sequence (Pb^2+^: 3.905 > Cr^3+^: 2.283 > Cd^2+^: 1.702 L mg^−1^), indicating progressively stronger surface binding affinity. These *K*_L_ values are characteristic of chemisorptive interactions, indicating relatively strong surface affinity compared with unmodified montmorillonite reported in the literature, which suggests that DES treatment generates surface sites of enhanced binding affinity.

The single-component *q*_max_ values exceed the equilibrium capacities from the mixed-metal system, with ratios of 56% (Pb^2+^), 57% (Cr^3+^), and 49% (Cd^2+^), consistent with competitive adsorption suppression in the multi-ion system. The fitted isotherm curves are presented in Fig. 9.

#### 3.5.1 Effect of DES modification on adsorption capacity

Table 3 quantifies the effect of DES treatment against an unmodified control measured under identical conditions. Single-component *q*_max_ increased by 158% for Pb^2+^, 163% for Cd^2+^, and 927% for Cr^3+^. The Cr^3+^ enhancement is the largest in relative terms partly because its baseline capacity on the untreated clay is very low (0.015 mg g^−1^), expressed in absolute terms, the Cr^3+^ gain (0.139 mg g^−1^) is of comparable magnitude to the Pb^2+^ gain (0.426 mg g^−1^) once scaled by the respective *K*_L_ values. This pattern is consistent with the surface chemistry established elsewhere in this work. DES treatment substantially redistributes and increases accessible surface hydroxyl groups without altering the underlying aluminosilicate framework, the hydroxyl-associated O 1s component grows at the expense of lattice oxygen while the XRD basal reflections remain unshifted, the point of zero charge rises from pH 2.5 to 3.2 as a result, and BET surface area increases moderately alongside it. The capacity gain is therefore best interpreted as evidence of enhanced site accessibility and binding affinity at existing hydroxyl positions, rather than the generation of an entirely new population of adsorption centres.

**Table 3:** Comparison of adsorption capacities of montmorillonite following DES modification

Target metal	*q*_max_ unmodified (mg g^−1^)	*q*_max_ DES-modified (mg g^−1^)
Pb^2+^	0.270	0.696
Cr^3+^	0.015	0.154
Cd^2+^	0.040	0.105

The trace-level initial metal concentrations used in the isotherm experiments (0.10–5.00 mg L^−1^, Section 2.5) were selected to characterize adsorption performance under conditions representative of real contaminated water, rather than the synthetic solutions spiked one to several orders of magnitude higher that are typically used in the literature to drive adsorption sites toward saturation. The correspondingly modest *q*_max_ values obtained here are a direct consequence of this design choice rather than a limitation of the DES-modified material, and this concentration effect is independent of, and additional to, the competitive-adsorption suppression discussed for the mixed-metal system in Section 3.2.

### 3.6 Recyclability

The recyclability of DES-modified montmorillonite was assessed over five adsorption–regeneration cycles using 0.1 M HNO_3_ as the regenerant. Capacity retention after five cycles was 87.2% for Pb^2+^, 74.2% for Cr^3+^, and 68.9% for Cd^2+^. The highest retention for Pb^2+^ is consistent with its stronger inner-sphere surface binding, while the more pronounced decline for Cd^2+^ reflects its weaker coordination and greater susceptibility to site loss under repeated acid treatment. The increasing cycle-to-cycle variability observed from cycle 3 onward is attributed to progressive surface heterogeneity arising from repeated HNO_3_ exposure. The maintenance of capacity above 68% for all three metals through five cycles confirms the practical regenerability of the modified material under repeated acidic conditions.

The cycle-by-cycle capacity data are presented in Fig. 10. The progressive decline in capacity is attributed to incomplete desorption of strongly bound metal species and minor structural degradation of the montmorillonite surface under repeated acidic conditions. The 0.1 M HNO_3_ regeneration protocol is simple, low-cost, and avoids the use of organic solvents, in line with the green chemistry principles motivating the use of DES in the synthesis of the material. The maintained structural integrity across five cycles provides additional confidence that the layered aluminosilicate framework is sufficiently robust for practical application.

**Fig. 10:**
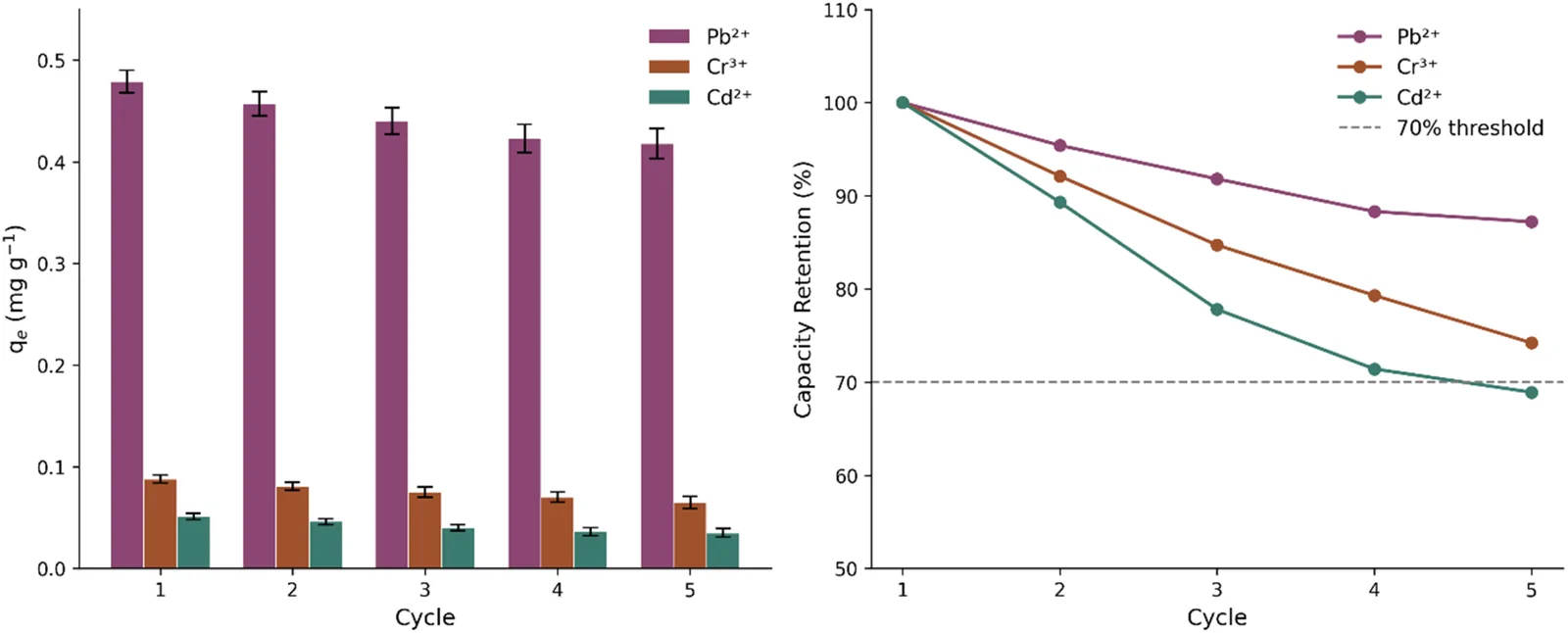
Recyclability of DES-modified montmorillonite over five adsorption–regeneration cycles.

### 3.7 Structural stability after metal incorporation

Post-adsorption characterization shows that the DES-modified montmorillonite retains its structural integrity following metal uptake. XRD patterns obtained after adsorption (Fig. 1c) show that the montmorillonite (02,11) reflection and the quartz/orthoclase reference peaks remain present at consistent positions, supporting preservation of the layered framework. FTIR spectra (Fig. 2) continue to display O–H and Si–O–Al vibrational bands, indicating that the key surface functionalities remain intact after metal coordination.

SEM post-adsorption (Fig. 4e and f) show that the platelet morphology characteristic of montmorillonite remains clearly visible. Although minor surface texturing is present, the overall particle architecture remains stable. SEM–EDS analysis shows that the montmorillonite retains its elemental composition, with silicon (Si), aluminum (Al), and sodium (Na) as the dominant elements, alongside minor contributions from iron (Fe), magnesium (Mg), and potassium (K). The absence of significant structural or compositional changes implies that the DES treatment, together with subsequent metal uptake, does not disrupt the primary aluminosilicate framework.

### 3.8 Electrochemical characterization of DES-modified montmorillonite

Electrochemical assessment was carried out to determine whether the structural changes established by XRD, FTIR, BET, and zeta potential analysis translate into a measurable change in interfacial charge-transfer behaviour. Cyclic voltammetry (CV) and electrochemical impedance spectroscopy (EIS) were used as complementary probes of electrochemical surface accessibility, with CV reporting the amplitude and reversibility of the redox response and EIS resolving the resistive and capacitive contributions to that response, providing a means to test whether the surface reconfiguration observed in the structural data carries a functional electrochemical signature.

#### 3.8.1 Cyclic voltammetric behavior

Cyclic voltammograms of the [Fe(CN)_6_]^3−/4−^ probe recorded on bare GCE, unmodified montmorillonite-modified GCE (MMT/GCE), and DES-modified montmorillonite-modified GCE (DES-MMT/GCE) are shown in Fig. 11a. The bare GCE produced a quasi-reversible redox couple with an anodic peak current (*I*_pa_) of 28.4 ± 0.3 µA and a peak-to-peak separation (Δ*E*_p_) of 90 mV. Deposition of unmodified montmorillonite suppressed this response, *I*_pa_ fell to 14.2 ± 0.3 µA and Δ*E*_p_ widened to 136 mV, suggesting that the poorly conductive clay layer partially blocking the electrode surface and slowing electron transfer to the probe.

**Fig. 11:**
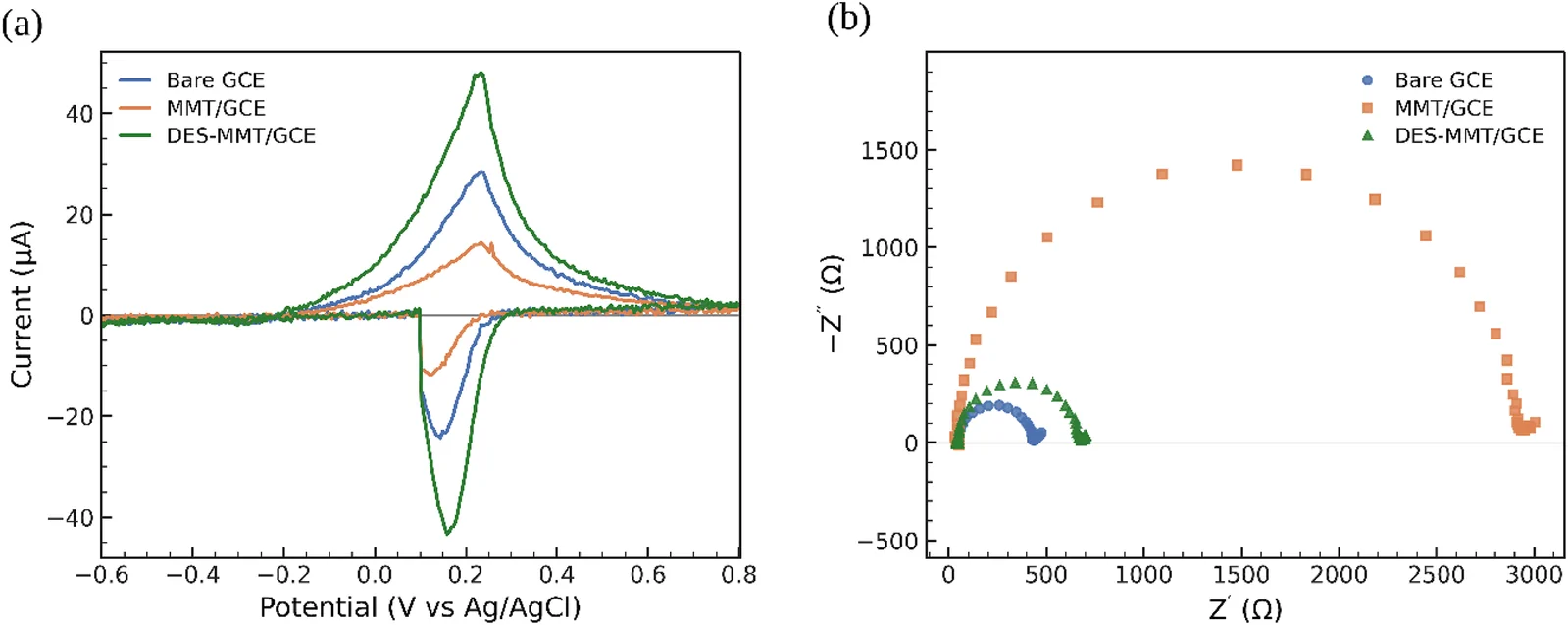
(a) Cyclic voltammograms, and (b) Nyquist plots of bare GCE, MMT/GCE, and DES-MMT/GCE.

DES-modified montmorillonite reversed this behaviour outright. *I*_pa_ increased to 47.6 ± 0.3 µA, a rise of 67.6% relative to bare GCE and 235.2% relative to unmodified MMT/GCE, while Δ*E*_p_ narrowed to 58 mV, close to the 59 mV limit expected for a fully reversible one-electron process at 25 °C. Because DES-MMT/GCE outperformed not only the clay-blocked MMT electrode but also the bare glassy carbon surface, DES treatment appears to do more than restore the electron-transfer pathway lost to the raw clay coating: it produces an interface with a greater density of accessible electroactive sites than the underlying GCE itself. A comparable recovery of redox activity on clay-modified electrodes following surface activation has been reported for phyllosilicate-based sensing platforms,^19^ though the enhancement observed here, an anodic peak current exceeding that of the bare electrode, is a comparatively strong outcome.

#### 3.8.2 Electrochemical impedance spectroscopy

Nyquist plots for the three electrodes (Fig. 11b) support the CV interpretation. Solution resistance (*R*_s_) was essentially constant across all conditions (44–47 Ω), confirming that electrolyte and cell geometry were unchanged and that the observed differences in impedance response originate at the electrode interface rather than in the bulk solution. Charge-transfer resistance (*R*_ct_) rose from 380 ± 4 Ω for bare GCE to 2850 ± 5 Ω for MMT/GCE, a 650% increase that mirrors the suppressed CV response and confirms that unmodified montmorillonite behaves as a resistive barrier to interfacial electron transfer.

DES-MMT/GCE reduced *R*_ct_ to 620 ± 9 Ω, a 78.2% decrease relative to MMT/GCE. Double-layer capacitance (*C*_dl_) increased in parallel, from 8.0 µF for MMT/GCE to 22.0 µF for DES-MMT/GCE, exceeding the bare-electrode value of 18.0 µF. This parallel with the 61% increase in BET surface area (42.3 to 68.1 m^2^ g^−1^) attributed to DES treatment (Section 3.1.4), and indicates that the additional surface area generated during modification is electrochemically accessible rather than confined to closed or non-conductive pore space. The Warburg coefficient followed the same trend, falling from 25.0 (MMT/GCE) to 10.0 Ω s^−0.5^ (DES-MMT/GCE), indicative of faster diffusive transport of the probe species through the modified layer.

The CV and EIS results converge on a single mechanistic picture: DES treatment does not simply compensate for the resistive penalty imposed by raw montmorillonite, it restructures the clay into a more open, better-connected framework that is both physically (BET) and electrochemically (*C*_dl_, *R*_ct_) more accessible than either the unmodified clay or the bare electrode. This result supports the characterization of montmorillonite as a structurally versatile but conductivity-limited scaffold for electrochemical materials once its native resistive character is addressed,^20^ and supports treating DES-modified montmorillonite as a candidate platform for clay-based electrode architectures rather than solely as an adsorbent.

## 4 Future perspectives

The present work establishes a mechanistic foundation for DES-mediated activation of layered aluminosilicates, and several directions of immediate priority emerge from the results.

The electrochemical findings warrant direct follow-up. The substantial reduction in charge-transfer resistance and the enhanced anodic response observed here were obtained without any optimisation of electrode preparation, binder composition, or DES treatment conditions. Systematic investigation of how treatment duration, ZnCl_2_ : urea molar ratio, and thermal parameters influence the electrochemical response of the modified montmorillonite would clarify the extent to which interfacial charge-transfer properties can be tuned. MSDES-modified montmorillonite is therefore a candidate support material in electrocatalytic systems, particularly for reactions that benefit from earth-abundant, hydroxyl-rich surfaces, including the oxygen evolution reaction (OER), hydrogen evolution reaction (HER), and non-precious-metal sensor platforms.

The zinc chloride–urea system used here represents one point in a broader compositional space. Metal salt-based DESs incorporating other transition metal chlorides, cobalt, nickel, copper, would introduce different coordination chemistries at the montmorillonite surface and potentially yield supports with distinct surface speciation and catalytic behaviour. Comparative studies across MSDES compositions, using the same montmorillonite host and characterization framework established here, would allow systematic mapping of how DES metal identity controls surface speciation and downstream functional behaviour.

The adsorption results also raise a selectivity question worth pursuing mechanistically. The pronounced preference for Pb^2+^ over Cr^3+^ and Cd^2+^ under competitive conditions, and the partial displacement of the latter two at longer contact times suggests that the DES-activated surface presents a non-uniform distribution of binding sites with differentiated affinity. Surface complexation modelling, ideally supported by X-ray photoelectron spectroscopy or extended X-ray absorption fine structure analysis, would allow direct assignment of binding geometries and oxidation state changes at the montmorillonite–metal interface, moving the interpretation beyond indirect spectroscopic inference.

Finally, the natural montmorillonite used here carries compositional heterogeneity inherent to its geological origin. Controlled studies using reference-grade homoionic montmorillonite would isolate the contribution of the DES treatment from variability in the starting material, providing a cleaner basis for mechanistic interpretation and improving reproducibility across laboratories.

## 5 Conclusion

This study demonstrates that treatment of natural sodium montmorillonite with a zinc chloride–urea metal salt-based deep eutectic solvent produces controlled structural reconfiguration of the layered aluminosilicate framework without compromising its crystallographic integrity. XRD, FTIR, SEM, XPS, zeta potential, and N_2_ physisorption data collectively show that DES exposure redistributes surface hydroxyl groups, increases accessible surface area by 61%, and generates an interfacial environment of enhanced metal coordination capacity, changes that are mechanistically distinct from those achievable through conventional acid treatment or pillaring. XPS provided direct quantitative evidence of zinc retention on the modified surface (Zn 2p, 0.74 atomic%, *versus* no detectable signal on unmodified clay), resolving the surface Zn content without invoking structural change to the aluminosilicate lattice.

The adsorption behaviour of Cr^3+^, Cd^2+^, and Pb^2+^ under realistic multi-ion, near-ambient concentration conditions reflects the selectivity hierarchy imposed by the DES-activated surface rather than bulk uptake capacity alone. Relative to unmodified montmorillonite measured under identical conditions, DES modification increased single-component *q*_max_ by 158% (Pb^2+^), 927% (Cr^3+^), and 163% (Cd^2+^), confirming that the enhancement stems from DES-induced surface changes rather than differences in test conditions. Langmuir isotherm fits indicate that surface coordination governs the ceiling of metal uptake, with Pb^2+^ showing the strongest affinity (*K*_L_ = 3.905 L mg^−1^, *q*_max_ = 0.696 mg g^−1^). Non-linear kinetic analysis further showed that Pb^2+^, Cr^3+^, and Cd^2+^ follow distinct rate-determining pathways, with Cr^3+^ and Cd^2+^ displaying signatures of progressive displacement by the more strongly bound Pb^2+^. Collectively, results support a mechanism in which DES-modified montmorillonite preferentially and durably retains Pb^2+^ while more weakly bound competing ions are displaced over extended contact time. Capacity retention above 87% for Pb^2+^ and above 68% for Cd^2+^ across five regeneration cycles confirms the practical durability of the modified material under repeated acidic conditions.

Electrochemical characterization provides complementary evidence that DES treatment enhances interfacial charge-transfer accessibility, reducing Rct by 78% and increasing anodic peak current by 235% relative to unmodified montmorillonite. These results suggest that the surface reconfiguration induced by MSDES treatment has implications beyond adsorption, extending to electrode interface engineering.

A mechanistic link between DES-induced structural modification and the metal-stabilising and electrochemical properties of the resulting material is demonstrated, alongside a reproducible, low-cost, and environmentally compatible preparation route for clay-based functional scaffolds. The results support further investigation of MSDES-modified montmorillonite in heterogeneous catalysis, sensor fabrication, and related interfacial applications.

## Author contributions

Bukar Lawan: Conceptualization, methodology, investigation, formal analysis, and writing original draft. Abubakar Shettima Kubri: Supervision, validation, writing, review and editing.

## Conflicts of interest

The authors declare no competing interests.

## Supplementary Material

RA-016-D6RA03601E-s001

## Data Availability

The data supporting this article have been included as part of the supplementary information (SI). Supplementary information is available. See DOI: https://doi.org/10.1039/d6ra03601e.
