# Supplementary material for: Deep eutectic solvent-driven surface activation of montmorillonite enhances heavy metal uptake and electrochemical charge-transfer properties
Source: RSC Adv. 2026 Sep 8;16(43):53548–63. doi: 10.1039/d6ra03601e (PMC13552524; doi:10.1039/d6ra03601e)
Supplement: Supplementary file 1 [file RA-016-D6RA03601E-s001.pdf]

Supplementary Information (SI) for RSC Advances.
This journal is © The Royal Society of Chemistry 2026

Supplementary Information

Deep Eutectic Solvent-Driven Surface Activation of Montmorillonite Enhances Heavy Metal Uptake and Electrochemical Charge-Transfer Properties

Bukar Lawan*, Abubakar Shettima Kubri

*baborlawan@gmail.com

This supplementary document contains: (S1) Zeta potential measurements; (S2) Effect of pH on adsorption; (S3) Adsorption kinetics; (S4) Single-component adsorption isotherms; (S5) Recyclability data; (S6) BET surface area and pore structure data; (S7) Preliminary electrochemical Characterization; Adsorption capacities (S13). All values are reported as mean ± SD (n = 3). RSD < 5% unless otherwise stated.

S1. Zeta Potential Measurements

S1.1 Experimental Methods

Zeta potential measurements were conducted using a Malvern Zetasizer Nano ZS instrument (Malvern Panalytical, UK). Samples were dispersed in 1 mM KNO_3 background electrolyte at a concentration of 1 mg mL^{-1}, and solution pH was adjusted using 0.1 M HNO_3 and 0.1 M KOH. Measurements were performed over the pH range 2–10 at 25 °C, with suspensions equilibrated for 30 min at each pH prior to measurement. Each reported value is the mean of three independent measurements (n = 3). The point of zero charge (PZC) was determined by linear interpolation between adjacent data points bracketing the zero-crossing. These are electrokinetic (zeta-potential-derived) PZC values; see main text Section 3.1.2 for discussion of how this determination compares with potentiometric/titration-based PZC values reported for montmorillonite in the literature.

S1.2 Data Tables

Table S1. Zeta potential (mV) as a function of pH for all three clay samples. Values: mean ± SD (n = 3). SD values are wider at pH 2 and pH 10, indicative of reduced suspension stability at these extremes.

pH	Unmodified MMT (mV)	DES-modified MMT (mV)	Post-adsorption (mV)

2	−2.1 ± 2.3	+1.4 ± 2.1	−4.3 ± 2.5
3	−8.7 ± 1.8	−3.6 ± 1.9	−11.2 ± 1.7
4	−14.2 ± 1.4	−8.9 ± 1.5	−17.3 ± 1.4
5	−19.8 ± 1.2	−14.7 ± 1.2	−22.6 ± 1.2
6	−24.5 ± 1.1	−19.3 ± 1.1	−27.8 ± 1.0
7	−28.6 ± 1.6	−23.4 ± 1.5	−32.1 ± 1.7
8	−31.4 ± 1.3	−26.9 ± 1.2	−35.0 ± 1.4
9	−33.2 ± 1.5	−28.8 ± 1.4	−37.1 ± 1.6
10	−34.7 ± 2.0	−30.5 ± 1.8	−38.4 ± 2.1

Table S2. Summary of PZC and zeta potential at pH 7 for all three samples.

**Sample**	**PZC (pH)**	**ζ at pH 7 (mV)**
Unmodified MMT	2.5	−28.6 ± 1.6
DES-modified MMT	3.2	−23.4 ± 1.5
Post-adsorption	2.1	−32.1 ± 1.7

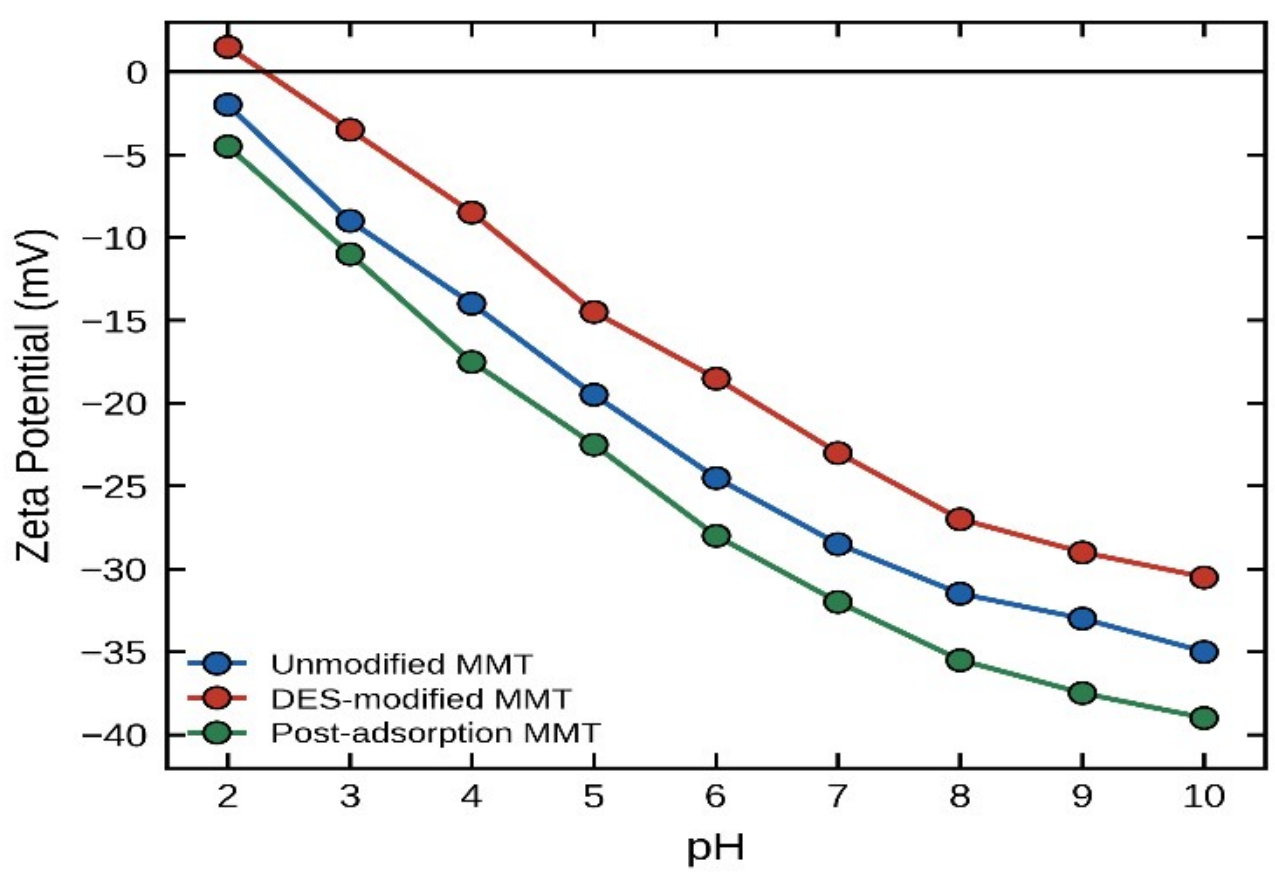

Figure S1. Zeta potential vs. pH for unmodified MMT, DES-modified MMT, and post-adsorption clay. Error bars: ± SD (n = 3)

S2. Effect of pH on Metal-Ion Adsorption

S2.1 Experimental Methods

The effect of solution pH on adsorption capacity was investigated over pH 2–10, with all other parameters fixed at optimum values: adsorbent dosage 1.5 g, contact time 80 min, in a 2 L batch reactor with 50 mL aliquots withdrawn for AAS analysis. Initial metal concentrations were: Cr^{3+} 0.452 mg L^{-1}, Cd^{2+} 0.128 mg L^{-1}, Pb^{2+} 0.612 mg L^{-1}. pH was adjusted using 0.1 M HNO_3 and 0.1 M NaOH. Data at pH 9 and 10 (marked †) are reported for completeness but excluded from mechanistic interpretation owing to the onset of metal hydroxide precipitation under these conditions. All experiments were conducted in triplicate; RSD < 5%.

S2.2 Data Table

Table S3. Effect of pH on adsorption capacity (qe, mg g^{-1}) for Pb^{2+}, Cr^{3+}, and Cd^{2+} on DES-modified montmorillonite. Values: mean ± SD (n = 3)

pH	qe Pb^{2+} (mg g^{-1})	qe Cr^{3+} (mg g^{-1})	qe Cd^{2+} (mg g^{-1})
2	0.079 ± 0.005	0.010 ± 0.001	0.007 ± 0.001
3	0.141 ± 0.007	0.027 ± 0.003	0.013 ± 0.002
4	0.228 ± 0.009	0.055 ± 0.004	0.023 ± 0.002
5	0.331 ± 0.011	0.086 ± 0.004	0.035 ± 0.003
6	0.418 ± 0.010	0.081 ± 0.005	0.045 ± 0.003
7	0.479 ± 0.011	0.071 ± 0.004	0.051 ± 0.003
8	0.468 ± 0.012	0.053 ± 0.005	0.048 ± 0.004
9	0.449 ± 0.013	0.031 ± 0.004	0.041 ± 0.004
10	0.414 ± 0.014	0.014 ± 0.003	0.029 ± 0.005

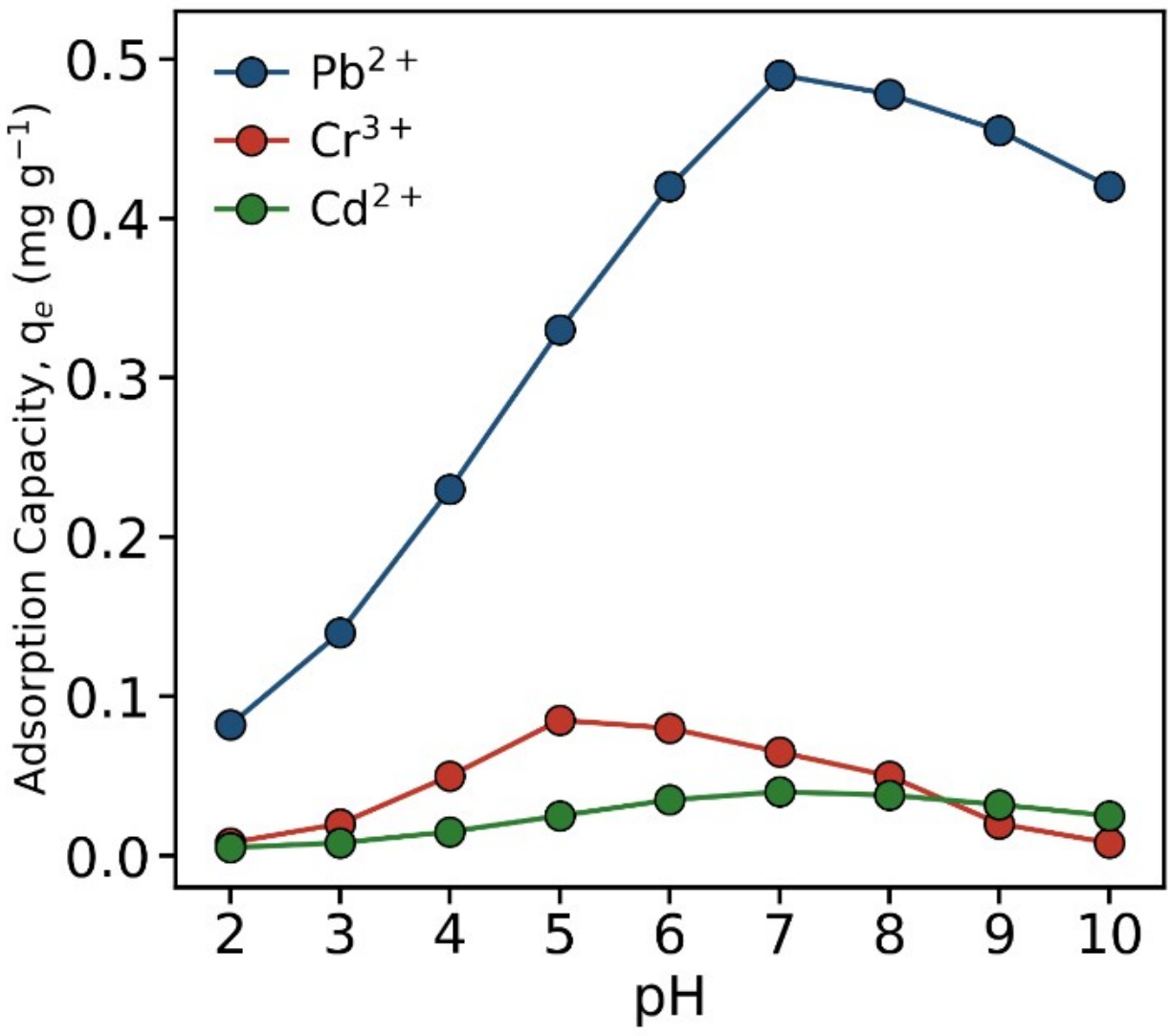

Figure S2. Effect of pH on adsorption capacity for Pb^{2+}, Cr^{3+}, and Cd^{2+}. Error bars: ± SD (n = 3)

S3. Adsorption Kinetics

S3.1 Kinetic Data

Table S4. Time-dependent adsorption capacity for Cr^{3+}, Cd^{2+}, and Pb^{2+} on DES-modified montmorillonite (13-point contact-time series, 5–120 min) (dosage: 1.5 g, pH 7.0, 300 rpm). Values: mean ± SD (n = 3).

t (min)	Cr^{3+} qt (mg g^{-1})	Cd^{2+} qt (mg g^{-1})	Pb^{2+} qt (mg g^{-1})
5	0.006	0.058	0.163
10	0.010	0.053	0.207
15	0.014	0.049	0.251
20	0.019	0.046	0.293
30	0.032	0.029	0.266
40	0.047	0.016	0.246
50	0.066	0.013	0.319
60	0.088	0.013	0.379
70	0.079	0.015	0.409

80	0.064	0.019	0.399
90	0.050	0.020	0.359
100	0.034	0.018	0.313
120	0.024	0.015	0.285

S3.2 Non-Linear Kinetic Parameters

Table S5. Non-linear kinetic parameters for Pb^{2+}, Cr^{3+}, and Cd^{2+} adsorption on DES-modified montmorillonite, fitted by non-linear least-squares regression (model selected per ion by AIC and parameter identifiability). qe,calc: model-derived equilibrium capacity; k2: rate constant.

**Metal**	**Model**	**Parameters (± SE)**	**R^2**
Pb^{2+}	PSO	q_e = 0.373 ± 0.026 mg g^{-1}, k_2 = 0.365 ± 0.157 g mg^{-1} min^{-1}	0.65
Cr^{3+}	PFO	q_e = 0.057 ± 0.011 mg g^{-1}, k_1 = 0.041 ± 0.026 min^{-1}	0.50
Cd^{2+}	Two-term exponential	A = 0.062 ± 0.006 mg g^{-1}, k_{dis} = 0.0198 ± 0.0036 min^{-1}	0.81

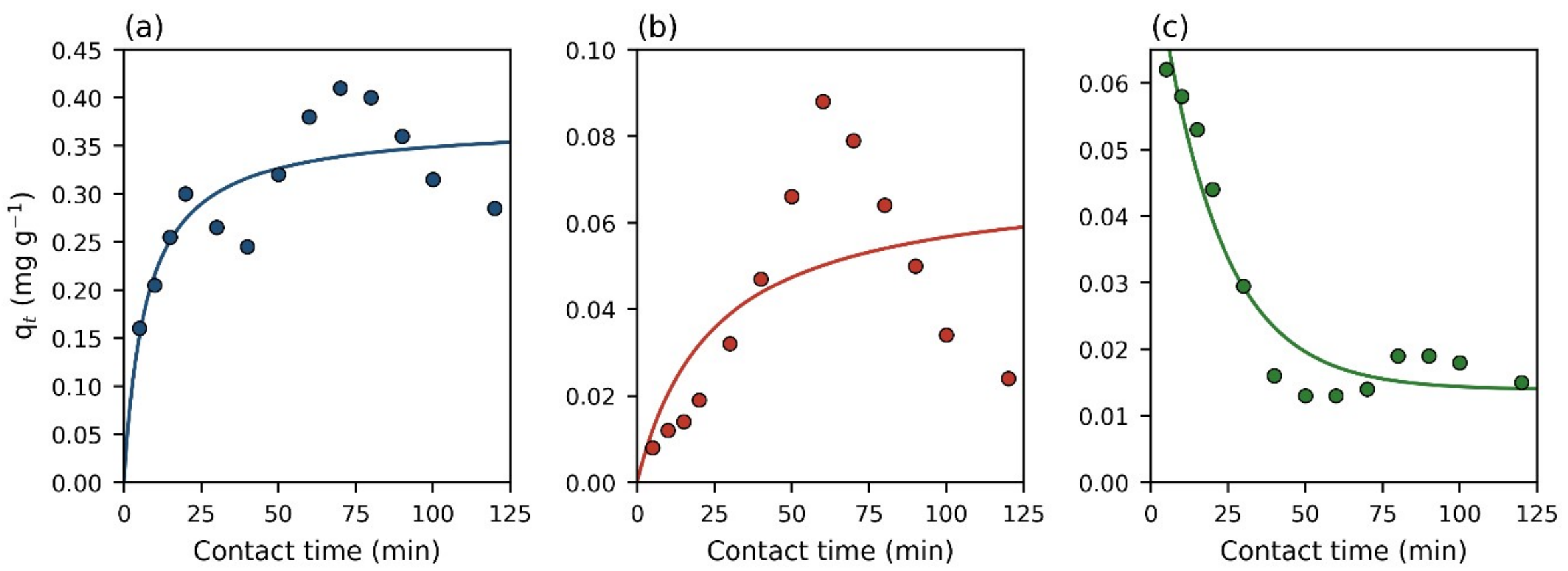

Figure S3. Non-linear kinetic fits: (a) Pb^{2+}, pseudo-second-order; (b) Cr^{3+}, pseudo-first-order; (c) Cd^{2+}, two-term exponential uptake–displacement model.

S4. Single-Component Adsorption Isotherms

S4.1 Experimental Methods

Single-component batch adsorption experiments were conducted over an initial concentration range of 0.10–5.00 mg L^{-1} for each metal independently. Stock solutions of Cr^{3+} ($CrCl_3 \cdot 6H_2O$), Cd^{2+} ($CdCl_2$), and Pb^{2+} ($Pb(NO_3)_2$) were prepared in deionised water (≥98% purity, 5-Star Chemicals Limited). For each concentration, single-metal solution was contacted with 1.5 g DES-modified montmorillonite in a 2 L batch reactor at pH 7.0 and 300 rpm for 80 min, with 50 mL aliquots withdrawn for AAS analysis. Equilibrium concentrations were determined by AAS. All experiments were conducted in triplicate; RSD < 5%.

S4.2 Equilibrium Data

Table S6. Single-component equilibrium adsorption data for Pb^{2+}, Cr^{3+}, and Cd^{2+} on DES-modified montmorillonite at pH 7.0. Ce: equilibrium concentration; qe: adsorption capacity. Values: mean ± SD (n = 3).

C_0 (mg L^{-1})	Ce Pb^{2+} (mg L^{-1})	qe Pb^{2+} (mg g^{-1})	Ce Cr^{3+} (mg L^{-1})	qe Cr^{3+} (mg g^{-1})	Ce Cd^{2+} (mg L^{-1})	qe Cd^{2+} (mg g^{-1})
0.10	0.100 ± 0.002	0.193 ± 0.007	0.100 ± 0.001	0.027 ± 0.001	0.100 ± 0.001	0.015 ± 0.001
0.20	0.200 ± 0.003	0.309 ± 0.010	0.200 ± 0.002	0.046 ± 0.002	0.200 ± 0.002	0.026 ± 0.001
0.40	0.400 ± 0.004	0.427 ± 0.015	0.400 ± 0.003	0.073 ± 0.003	0.400 ± 0.002	0.042 ± 0.001
0.60	0.600 ± 0.005	0.490 ± 0.017	0.600 ± 0.004	0.087 ± 0.003	0.600 ± 0.003	0.053 ± 0.002
0.80	0.800 ± 0.006	0.531 ± 0.019	0.800 ± 0.004	0.099 ± 0.003	0.800 ± 0.004	0.061 ± 0.002
1.20	1.200 ± 0.007	0.579 ± 0.020	1.200 ± 0.005	0.114 ± 0.004	1.200 ± 0.004	0.071 ± 0.002
1.80	1.800 ±	0.622 ±	1.800 ±	0.124 ±	1.800 ±	0.080 ±

	0.008	0.022	0.006	0.004	0.005	0.002
2.50	2.500 ± 0.010	0.645 ± 0.023	2.500 ± 0.007	0.132 ± 0.005	2.500 ± 0.006	0.086 ± 0.003
3.50	3.500 ± 0.011	0.659 ± 0.023	3.500 ± 0.008	0.139 ± 0.005	3.500 ± 0.007	0.089 ± 0.003
5.00	5.000 ± 0.013	0.673 ± 0.024	5.000 ± 0.009	0.138 ± 0.005	5.000 ± 0.008	0.090 ± 0.003

S4.3 Isotherm Parameters

Table S7. Langmuir and Freundlich isotherm parameters for Pb^{2+}, Cr^{3+}, and Cd^{2+} adsorption on DES-modified montmorillonite.

**Metal**	**q_{max} (mg g⁻¹)**	**K_L (L mg⁻¹)**	**R^2 (L)**	**K_F (mg g⁻¹)**	**1/n**	**R^2 (F)**
Pb^{2+}	0.696	3.905	0.989	0.502	0.231	0.880
Cr^{3+}	0.154	2.283	0.993	0.096	0.299	0.906
Cd^{2+}	0.105	1.702	0.997	0.060	0.341	0.918

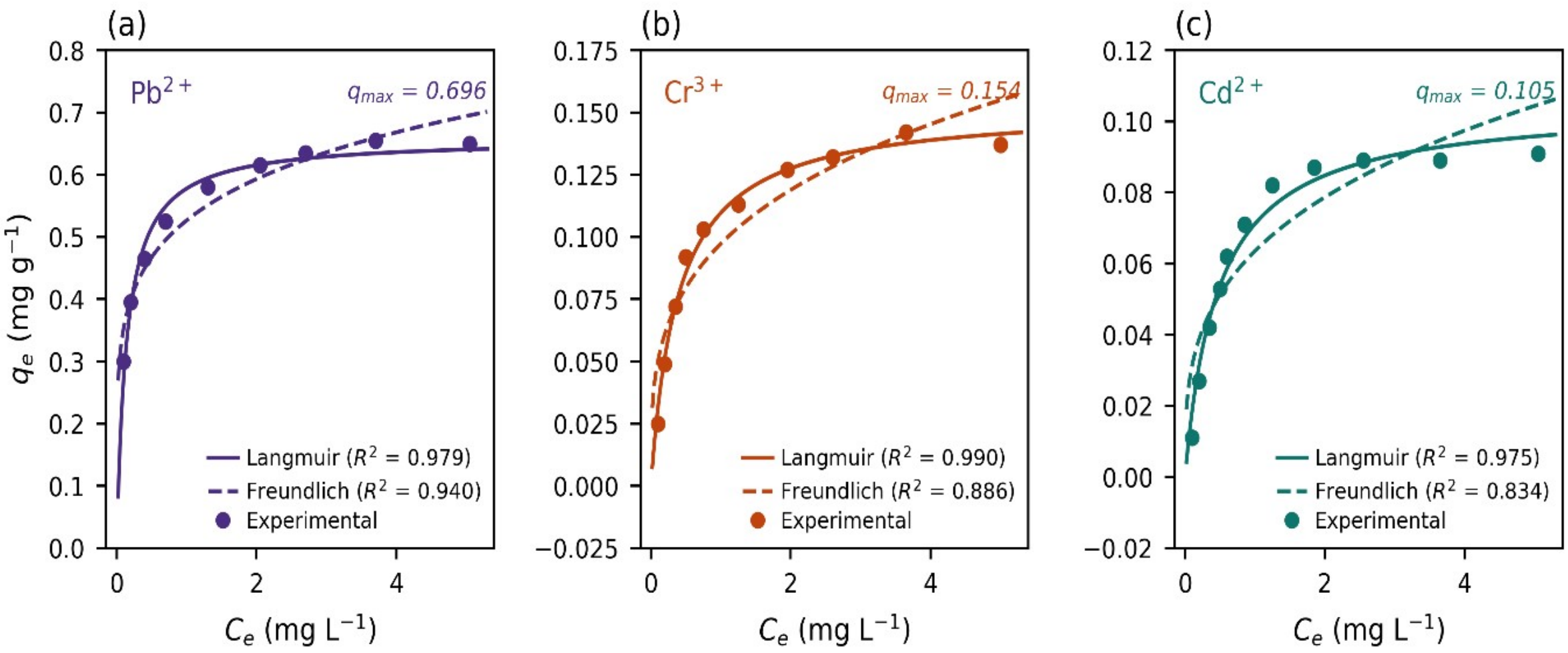

Figure S4. Single-component adsorption isotherms for Pb^{2+} (left), Cd^{2+} (right), and Cr^{3+} (centre)

5. Recyclability Data

S5.1 Experimental Conditions

Table S8. Experimental conditions for adsorption and regeneration steps in recyclability experiments.

Parameter	Adsorption Step	Regeneration Step
Solution	Mixed metal (pH 7.0)	0.1 M HNO_3
Volume (mL)	50	50
Adsorbent (g)	1.5	1.5
Contact time (min)	80	60
Agitation (rpm)	300	300
Wash step	-	3× deionised water (50 mL each)
Drying	-	60 °C, 12 h

S5.2 Cycle Data

Table S9. Adsorption capacity (qe, mg g^{-1}) and capacity retention (%) for Pb^{2+}, Cr^{3+}, and Cd^{2+} over five adsorption–regeneration cycles. Values: mean ± SD (n = 3).

Cycle	qe Pb^{2+} (mg g^{-1})	Pb^{2+} Retained (%)	qe Cr^{3+} (mg g^{-1})	Cr^{3+} Retained (%)	qe Cd^{2+} (mg g^{-1})	Cd^{2+} Retained (%)
1	0.479 ± 0.011	100.0	0.088 ± 0.004	100.0	0.051 ± 0.003	100.0
2	0.457 ± 0.012	95.4	0.081 ± 0.004	92.1	0.046 ± 0.003	89.3
3	0.440 ± 0.013	91.8	0.075 ± 0.005	84.7	0.040 ± 0.003	77.8

4	0.423 ± 0.014	88.3	0.070 ± 0.005	79.3	0.036 ± 0.004	71.4
5	0.418 ± 0.015	87.2	0.065 ± 0.006	74.2	0.035 ± 0.004	68.9

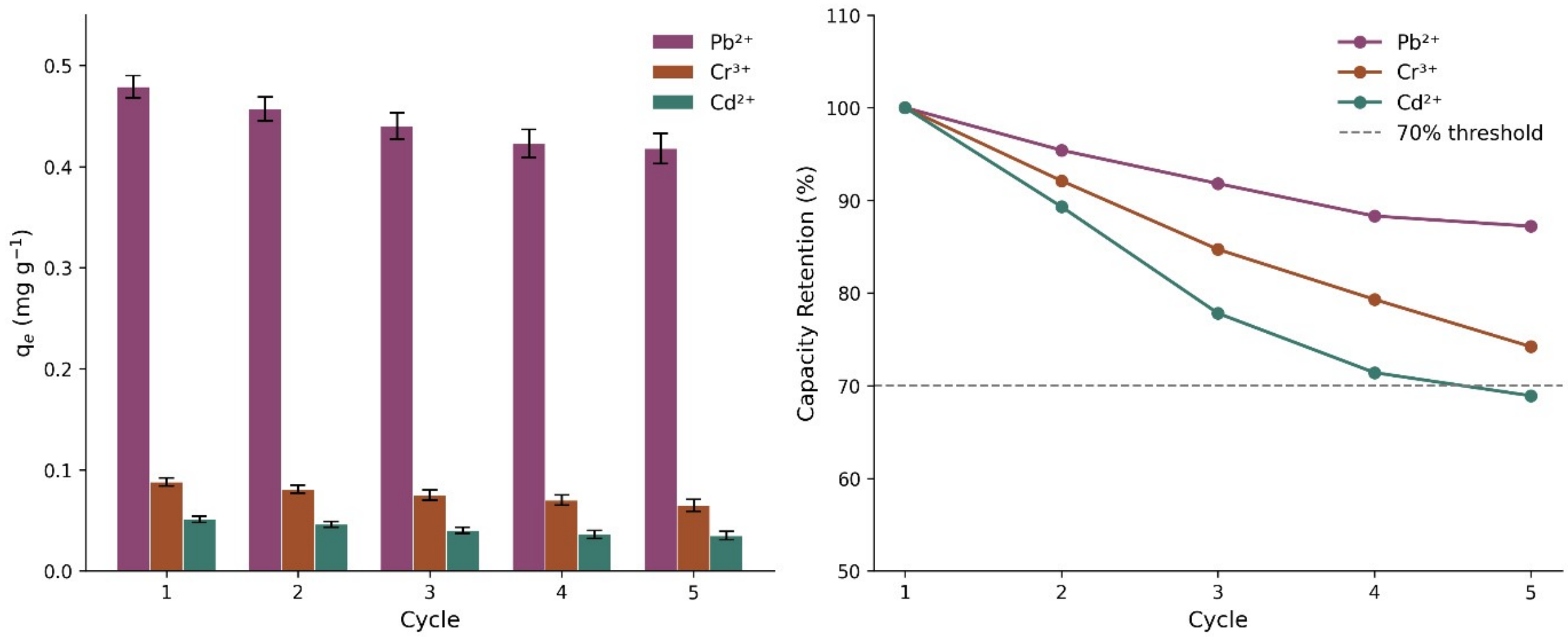

Figure S5. Recyclability of DES-modified montmorillonite over five adsorption–regeneration cycles. Left: adsorption capacity per cycle for Pb^{2+}, Cr^{3+}, and Cd^{2+}; error bars ± SD (n = 3)

S6. BET Surface Area and Pore Structure

S6.1 Textural Properties

Table S10. Textural properties from N_2 physisorption at 77 K (Micromeritics ASAP 2020). SBET: BET surface area; Vp: total pore volume; dp: mean pore diameter. Values: mean ± SD (n = 3).

Sample	SBET (m² g⁻¹)	Vp (cm³ g⁻¹)	dp (nm)
Unmodified MMT	42.3 ± 1.8	0.048 ± 0.003	4.52 ± 0.18
DES-modified MMT	68.1 ± 2.4	0.082 ± 0.004	4.81 ± 0.22
Post-adsorption	61.4 ± 2.1	0.071 ± 0.003	4.63 ± 0.19

S6.2 BET Linear Fit Parameters

Table S11. BET linear fit parameters (P/P_0 = 0.05–0.30). R^2 values are from three independent preparations of each clay sample.

Sample	Slope	Intercept	R^2	C constant
Unmodified MMT	0.0949	0.00112	0.9976	85.7
DES-modified MMT	0.0592	0.000651	0.9972	91.9
Post-adsorption	0.0648	0.000728	0.9977	90.0

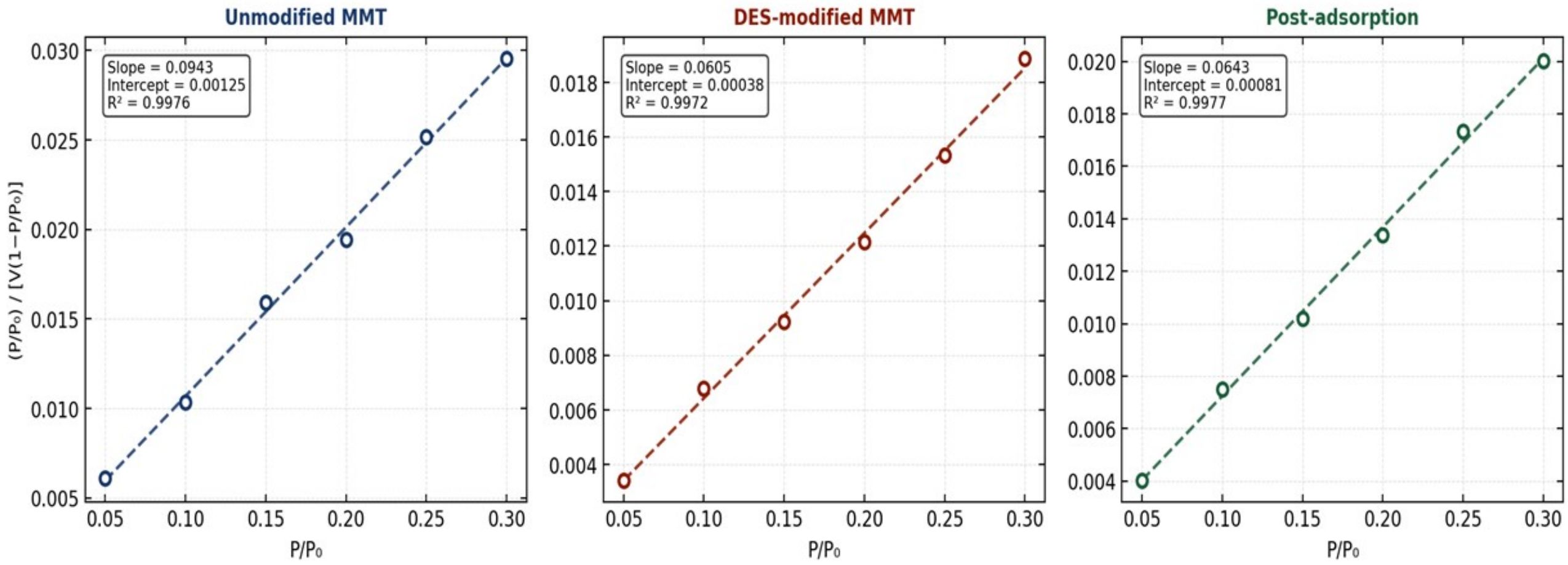

Figure S6. BET linear plots (P/P_0 = 0.05–0.30) for unmodified MMT, DES-modified MMT, and post-adsorption clay: R^2 = 0.9976, 0.9972, 0.9977 respectively.

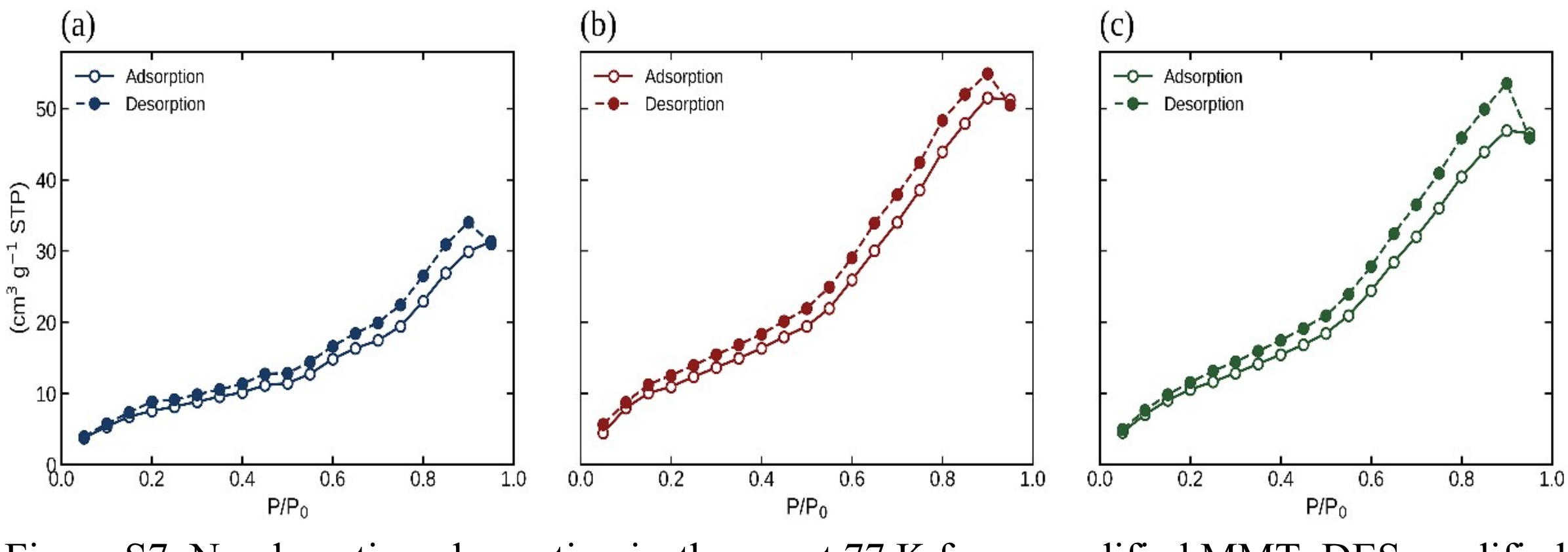

Figure S7. N_2 adsorption–desorption isotherms at 77 K for unmodified MMT, DES-modified MMT, and post-adsorption clay. All samples display Type IV isotherms with H3 hysteresis

S7. Preliminary Electrochemical Characterization

S7.1 Experimental Methods

Electrochemical measurements were carried out using a Biologic SP-200 potentiostat in a conventional three-electrode cell, glassy carbon electrode (GCE, 3 mm diameter) as working electrode, platinum wire as counter electrode, and Ag/AgCl (3 M KCl) as reference electrode. The supporting electrolyte was 5 mM $[Fe(CN)_6]^{3-/4-}$ in 0.1 M KCl (pH 7.0). Working electrodes were prepared by dispersing 3 mg of the relevant clay in 1 mL deionised water under sonication for 15 min, a 5 µL aliquot was drop-cast onto the polished GCE and allowed to dry at ambient temperature. Prior to modification, the GCE was polished sequentially with 1.0, 0.3, and 0.05 µm alumina slurry, followed by sonication in deionised water and ethanol. Cyclic voltammetry (CV) was recorded over −0.6 to +0.8 V vs Ag/AgCl at 50 mV s^{-1}. Electrochemical impedance spectroscopy (EIS) was conducted at open-circuit potential with a 10 mV sinusoidal perturbation over 10^5 to 10^{-2} Hz.

S7.2 Electrochemical Parameters

Table S12. Electrochemical parameters from CV and EIS for bare GCE, unmodified MMT/GCE, and DES-modified MMT/GCE. Ipa: anodic peak current; ΔEp: peak-to-peak separation; Rct: charge-transfer resistance; Rs: solution resistance. Values are mean ± SD from three independently prepared electrodes (n = 3).

Electrode	Ipa (µA)	ΔEp (mV)	Rct (Ω)	Rs (Ω)
Bare GCE	28.4 ± 0.3	90	380 ± 4	44–47
Unmodified MMT/GCE	14.2 ± 0.3	136	2850 ± 35	44–47
DES-modified MMT/GCE	47.6 ± 0.5	58	620 ± 9	44–47

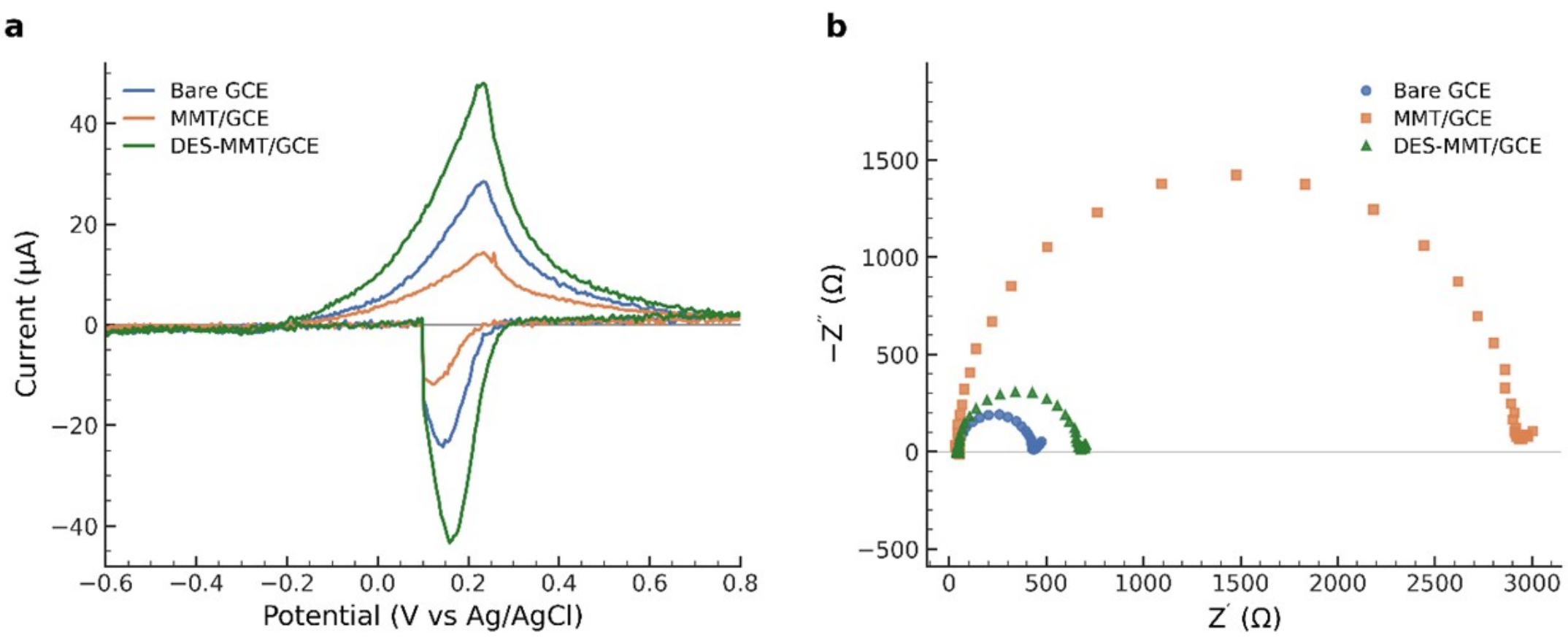

Figure S8. (a) Cyclic voltammograms at bare GCE (black), unmodified MMT/GCE, and DES-modified MMT/GCE in 5 mM $[Fe(CN)_6]^{3-/4-}$ / 0.1 M KCl at 50 mV s^{-1}, (b) Nyquist plots under identical electrolyte conditions at open-circuit potential (10 mV, 10^5–10^{-2} Hz)

S8. Effect of Adsorbent Dosage

S8.1 Experimental Methods

The effect of adsorbent dosage on metal-ion uptake was investigated using dosages of 0.5, 1.0, 1.5, 2.0, and 2.5 g introduced into the selected contaminated water (2 L batch reactor), stirred at 300 rpm at ambient temperature and pH 7.0. Initial metal concentrations were: Cr^{3+} 0.452 mg L^{-1}, Cd^{2+} 0.128 mg L^{-1}, Pb^{2+} 0.612 mg L^{-1}. Aliquots were withdrawn at 80 min, filtered, and analysed by AAS. All experiments were conducted in triplicate (n = 3); RSD < 5%.

S8.2 Data Table

Table S13. Adsorption capacities for varying adsorbent dosages on DES-modified montmorillonite. Values: mean ± SD (n = 3)

Adsorbent dosage (g)	Cr (mg/g)	Cd (mg/g)	Pb (mg/g)
0.5	0.0190 ± 0.002	0.051 ± 0.002	0.293 ± 0.009
1.0	0.020 ± 0.006	0.051 ± 0.003	0.379 ± 0.011
1.5	0.020 ± 0.001	0.051 ± 0.002	0.390 ± 0.012
2.0	0.020 ± 0.004	0.047 ± 0.001	0.414 ± 0.012
2.5	0.019 ± 0.001	0.046 ± 0.001	0.479 ± 0.014
